# Investigating social comparison behaviour in an immersive virtual reality classroom based on eye-movement data

**DOI:** 10.1038/s41598-023-41704-2

**Published:** 2023-09-06

**Authors:** Lisa Hasenbein, Philipp Stark, Ulrich Trautwein, Hong Gao, Enkelejda Kasneci, Richard Göllner

**Affiliations:** 1 Hector Research Institute of Education Sciences and Psychology, University of Tübingen, Europastraße 6, 72072 Tübingen, Germany; 2 Human-Centered Technologies for Learning, Technical University of Munich, Arcisstraße 21, 80333 Munich, Germany

**Keywords:** Psychology, Human behaviour, Psychology and behaviour

## Abstract

Higher-achieving peers have repeatedly been found to negatively impact students’ evaluations of their own academic abilities (i.e., Big-Fish-Little-Pond Effect). Building on social comparison theory, this pattern is assumed to result from students comparing themselves to their classmates; however, based on existing research designs, it remains unclear how exactly students make use of social comparison information in the classroom. To determine the extent to which students (*N* = 353 sixth graders) actively attend and respond to social comparison information in the form of peers’ achievement-related behaviour, we used eye-tracking data from an immersive virtual reality (IVR) classroom. IVR classrooms offer unprecedented opportunities for psychological classroom research as they allow to integrate authentic classroom scenarios with maximum experimental control. In the present study, we experimentally varied virtual classmates’ achievement-related behaviour (i.e., their hand-raising in response to the teacher’s questions) during instruction, and students’ eye and gaze data showed that they actively processed this social comparison information. Students who attended more to social comparison information (as indicated by more frequent and longer gaze durations at peer learners) had less favourable self-evaluations. We discuss implications for the future use of IVR environments to study behaviours in the classroom and beyond.

## Introduction

Social comparisons are a central aspect of human nature. How we perceive and evaluate ourselves (e.g., how competent we think we are in a specific domain) is substantially shaped by who we compare ourselves with^1^. Understanding these social comparison processes in-depth is crucial, considering that the beliefs we hold about ourselves and our abilities have far-reaching consequences for individual life trajectories. In particular, a high academic self-concept—meaning high levels of confidence in one’s own abilities and performance at school—is considered a critical determinant of successful learning and a fundamental prerequisite for achieving one’s academic goals as well as successful education and career choices^2–4^.

It is thus no wonder that the determinants of students’ academic self-concept—in other words, questions of what leads to individual differences in beliefs about one’s own abilities—are among the most studied phenomena in social and educational psychology^5,6^. One of the most prominent findings in the educational context, the so-called Big-Fish-Little-Pond Effect (BFLPE)^7^, suggests that equally able students will give worse evaluations of their own academic abilities when in a high-performing class than when in a class with a lower average performance level. The BFLPE has been confirmed for different grade levels and school types and in different countries around the world^4,8^. BFLPE research typically sets a class’s performance level in relation to individual students’ performance; if classmates’ performance is significantly negatively related to individual students’ academic self-concept, it is concluded that social comparisons have occurred (i.e., lower achieving students have worse self-evaluations of their academic abilities due to comparisons with higher achieving peers).

A question that has been rather tangential to BFLPE research so far but is central to understanding its underlying processes pertains to the nature of the social comparisons that take place: Do students in fact *actively compare* themselves with their peers during instruction? Or are students’ self-evaluations instead affected by explicit performance feedback from peers, teachers, or grades?

To move beyond the status quo of research on the BFLPE and associated social comparisons, the present study brings together three strands of research, which we explain in more detail in the following sections. First, we outline the role of active social comparison behaviour in the classroom for students’ self-evaluations as a central open question from an educational research perspective. Second, we draw on eye-tracking research and describe how eye movements can be used to obtain indicators of social comparison behaviour in general and specifically in IVR settings. To this end, we thirdly illustrate how immersive virtual reality (IVR) provides the optimal experimental setting for such research, as it (a) allows the realistic and authentic simulation of a social classroom scenario and (b) makes it possible to collect fine-grained process data in a controlled environment.

In a typical classroom situation, peer learners are considered the most important source of information and the primary reference group for social comparisons^5,9^. The large body of BFLPE research is based on correlational analyses of large-scale data from real-world learning contexts. Therefore, although this literature contains compelling evidence^10,11^, such studies have yet to identify the actual causes and underlying mechanisms^12^. To gain respective insights, researchers would need to observe social comparison processes at the exact moment when they occur—something that is difficult to achieve in real-world classrooms given all the complex dynamics and simultaneous events during instruction^13^. To achieve standardised conditions that can allow such fine-grained insights into social comparison processes and help answer questions about causality, social psychological studies typically take a strictly experimental approach and therefore tend to be situated in lab settings^14,15^. Typically, these experiments provide manipulated performance-related social comparison information (i.e., researchers explicitly tell participants that they belong to the lowest vs. highest achieving group)^16,17^ or instruct participants to compare themselves with a specific (fictitious) comparison target (i.e., participants do not need to actively search for social comparison information)^18–20^. As a result of these experimental designs, such studies cannot answer the question of whether students in an actual—much more complex and dynamic—classroom situation actively engage in social comparisons themselves or are rather passively affected by comparisons stemming from their peers, teacher, or grades^21^. Notably, all of the aforementioned research has relied on students’ self-reports to gain insights into social comparison processes and the resulting differences in academic self-evaluations^22–24^. Hence, their findings ultimately rely on students’ introspective statements, which are likely to differ in the extent to which they correspond to actual behaviour^25^.

Eye-tracking offers great potential with respect to gaining a more in-depth and objective understanding of the processes underlying social comparisons^26,27^. Aggregated eye and gaze features can be used to identify the different mechanisms behind social comparison behaviour. First, students need to orient themselves in a classroom situation and notice the social comparison information. Gaze data show where and when students look at particular objects in the classroom^28^. Given that humans are able to guide their attention in the world and selectively focus on relevant objects while ignoring others, it is assumed that moving one’s eyes to a relevant location in space is an indication that one is paying attention to the information contained in the object of one’s gaze (i.e., overt spatial or so-called visual attention)^29^. Consequently, by looking at a high number of peer learners, students show that they have noticed the corresponding social comparison information in the classroom. Second, beyond simply noticing this information, students need to actively make social comparisons by processing the social comparison information. Hence, not only looking at a higher number of peer learners but also the frequency and duration of such gazes provide valuable insights^30^. Based on what is known about fixations (i.e., the time a gaze rests at a particular place), more frequent and longer visual attention are a sign that the object that the student looked at is being processed more deeply^31,32^. In other words, the more often and the longer students look at their classmates, the more they are presumably processing the provided social comparison information (i.e., consider it when making comparisons). Lastly, pupillometry provides further insights into students’ responses to the social comparison information. Pupil diameter has been associated with cognitive load^33,34^, information encoding and retrieval^35–37^, and affective arousal^38–40^. In this vein, greater pupil diameter indicates that students are more concerned with the (social comparison) information they process, both cognitively (e.g., because the information is new and might deviate from their usual experiences) and affectively (e.g., because students relate the information to themselves and use it to compare their own abilities).

**Figure 1:**
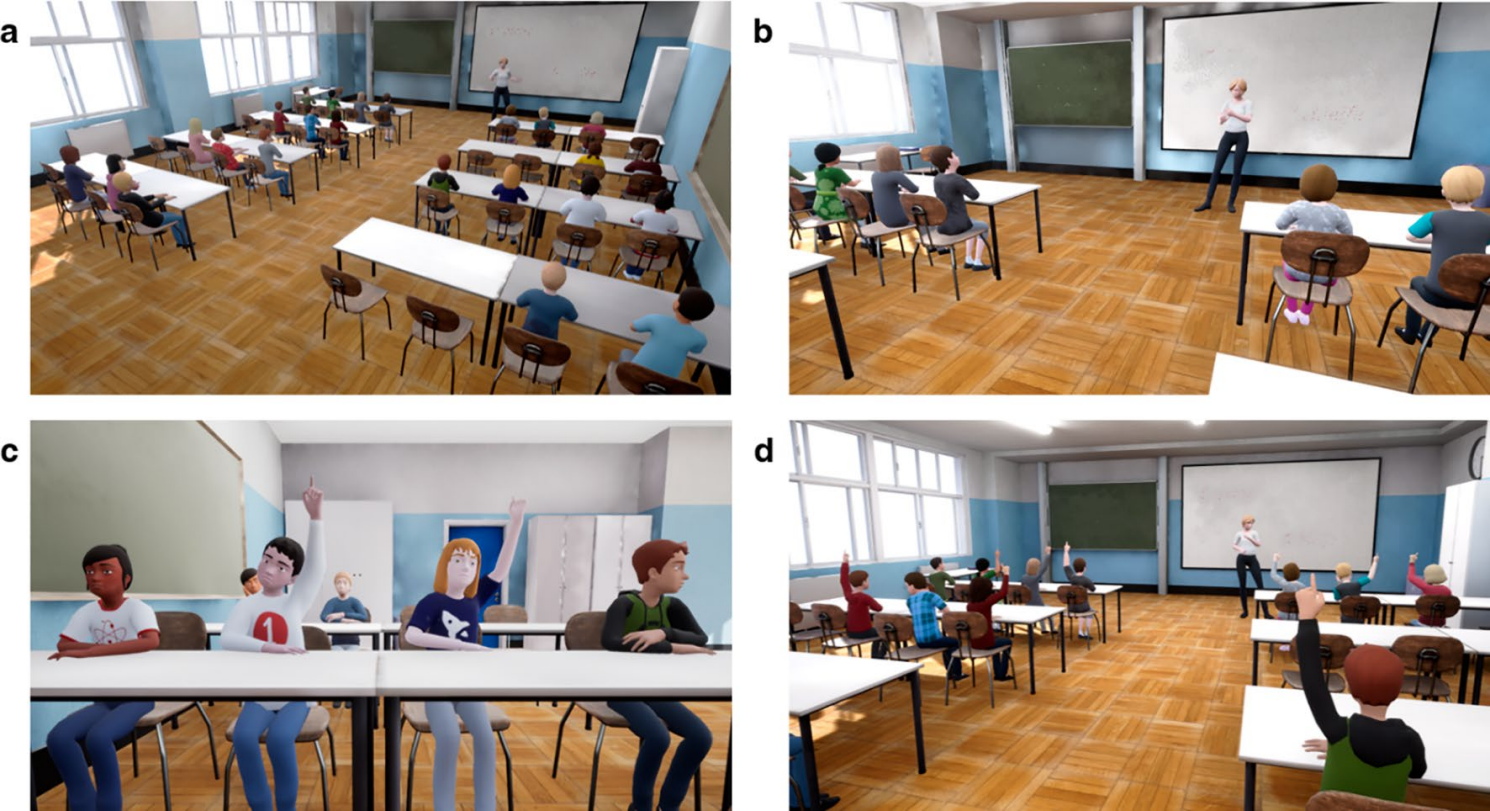
Virtual classroom situation with different peer behaviours. The images show (**a**) a bird’s eye view of the IVR classroom, and (**b**) the view of a student in a sitting position in the second row from the front (both in a situation without any hand-raising by peers), (**c**) a close-up of virtual peer learners’ hand-raising, and (**d**) a situation with 80% hand-raising peer learners from the perspective of a student sitting in the back.

To gain insights into actual social comparison behaviour during instruction, we used an IVR classroom in our study (see Fig. 1). Recently, motivated by continuous advances in IVR technology, increasing numbers of researchers have come to acknowledge the methodological affordances of IVR as an experimental tool^41,42^. IVRs—once programmed—provide cost- and time-efficient, highly reproducible testing settings with maximum control of confounding and manipulated variables while simultaneously providing an authentic experience (for examples in the classroom context, see Refs.^43–45^). Advocates of IVR as an experimental tool highlight evidence that users’ behaviour in IVR settings is similar to real-life behaviour^46,47^. Children in particular have been found to experience high levels of immersion and an exhaustive sense of presence in IVR environments^48^. Here, presence refers to (a) a spatial perception of actually *being in* the virtual environment^49,50^, and (b) a social perception of *being with another* in the virtual environment and a respective response to and/or interaction with virtual actors^51,52^. In addition to IVR providing authentic and yet experimentally controlled research set-ups, modern head-mounted displays (HMDs) with integrated eye-tracking devices simultaneously make it possible to collect eye-tracking data nonintrusively and under standardised (lighting) conditions^53^. Consequently, IVR makes it easy to examine behavioural data such as pupillometry or visual attention as a complement to commonly used self-report measures. Using eye tracking as an additional source of information can provide a more in-depth and unbiased understanding of the processes underlying social comparisons^26,27^.

Taken together, the present study aims to advance research on social comparisons in the classroom by using an IVR environment as an authentic but experimentally controllable set-up. More specifically, we manipulated social comparison information (i.e., virtual peer learners’ achievement-related behaviour) in an IVR classroom (see Fig. 1) to systematically examine social comparison behaviour in the classroom and the respective effects on students’ academic self-evaluations. We implemented four different performance levels for the virtual peer learners in the IVR classroom by systematically varying the proportion of virtual peer learners who actively participated and raised their hands to indicate that they knew the correct answer. To examine the extent to which students actively compared themselves with their peers (rather than being affected by explicit performance feedback from peers, teachers, or grades), we chose hand-raising as a performance-related behaviour that (a) naturally occurs during classroom learning, (b) was suitable in the context of the experiment from a technical implementation perspective, and (c) was obvious for participating students to observe (without being too explicit). To emphasise that hand-raising was an indicator of performance, the teacher’s questions in the IVR lesson had a certain level of difficulty (e.g., they referred to new content, they required knowledge transfer and application). In addition, the virtual classmates who raised their hands and were called on always provided correct and thorough answers to the teacher's questions so that the performance-related attributions of students’ hand-raising behaviour would be more salient. In the four conditions, 20% vs. 35% vs. 65% vs. 80% of the virtual peer learners exhibited high-achieving hand-raising behaviour. The participating students were told they would experience a simulation of a real-world classroom scenario in the IVR.

To gain insights into the underlying social comparison processes, we used eye-tracking data from the IVR classroom to examine (1) the extent to which students attend and respond to peer learners’ achievement-related behaviour and (2) how students’ behavioural responses to the provided social comparison information are related to differences in their self-concept. We used four eye-tracking features as indicators of active social comparison behaviour: The *number of peer learners looked at* (i.e., the extent to which students noticed social comparison information), the *frequency of gazing at peer learners* (i.e., how often students’ visual attention shifted to social comparison information over the course of the lesson), the *total time spent gazing at peer learners* (i.e., how long students spent processing the social comparison information), and students’ *mean pupil diameter* (i.e., reflecting cognitive and affective arousal associated with information processing). We asked:

1. Do the experimental variations in virtual peer learners’ hand-raising behaviour affect students’ visual attention to virtual peers (i.e., the number of peers looked at, the frequency of gazing at peers, and total time spent gazing at peers) and their pupil diameters?
2. Are the eye and gaze features as indicators of active social comparison behaviour in the IVR classroom related to students’ situational self-concept?

## Results

We first examined to what extent virtual peer learners’ achievement-related behaviour (i.e. the experimental variation of hand-raising behaviour) affects how students attend and respond to this social comparison information provided in the IVR classroom. We investigated how the different hand-raising conditions (20% vs. 35% vs. 65% vs. 80% of classmates raising their hands and therefore engaging in high-achieving behaviour) impact students’ visual attention to virtual peer learners (i.e., the number of peer learners looked at, the frequency of gazing at peers and the total gaze time on them) as well as students’ mean pupil diameter (as an indicator of overall arousal associated with processing activities).

### More visual attention to peer learners and greater pupil diameter in extreme hand-raising conditions

Whereas we expected higher levels of visual attention to the virtual classmates and increased pupil diameter as the proportion of peer learners raising their hands increased, descriptive statistics for the eye-movement features in the experimental hand-raising conditions (see Table 1) showed the highest values for both visual attention to virtual classmates and pupil diameter in the conditions in which 20% and 80% of peer learners raised their hands. This finding indicates that students were particularly likely to notice and respond to their peers’ implicit achievement-related (i.e., hand-raising) behaviour when the respective social comparison information could be clearly interpreted and a clear minority/majority of peer learners engaged in high-achieving behaviour.

**Table 1:** Descriptive statistics for eye-movement features in different hand-raising conditions.

Experimental condition	Number of peers looked at		Frequency of gazing at peers (log)		Total time gazing at peers (log)		Mean pupil diameter (log)	
	*M*	*SD*	*M*	*SD*	*M*	*SD*	*M*	*SD*
20% hand-raising	5.56	2.81	3.38	1.19	3.41	1.35	− 0.12	0.12
35% hand-raising	5.48	2.88	3.23	1.15	3.28	1.29	− 0.12	0.12
65% hand-raising	5.05	2.65	3.09	1.11	3.09	1.18	− 0.10	0.14
80% hand-raising	6.10	2.64	3.53	1.10	3.57	1.20	− 0.05	0.14

Hand-raising refers to the experimental manipulation of peer learners’ performance level via the proportion of hand-raising students. Participants (*N* = 353) were randomly assigned to one of the four hand-raising conditions, with 20% (*n* = 92), 35% (*n* = 86), 65% (*n* = 85) and 80% (*n* = 90) of peer learners raising their hands, respectively.

We analysed the effects of the four hand-raising conditions on the four indicators of social comparisons in more detail via multiple regression models. We included a number of covariates in the models to account for (a) individual differences in the social comparison context (e.g., gender, individual competence beliefs) as well as (b) potential effects of the IVR classroom configuration (field of view, avatar visualisation style) on the processing of social comparison information in the IVR classroom. Detailed information on all of the covariates is included in the Method section and in the Supplementary Appendices 1–3.

### Proportion of hand-raising peer learners affects how many peer learners are looked at but not for how often or how long

**Figure 2:**
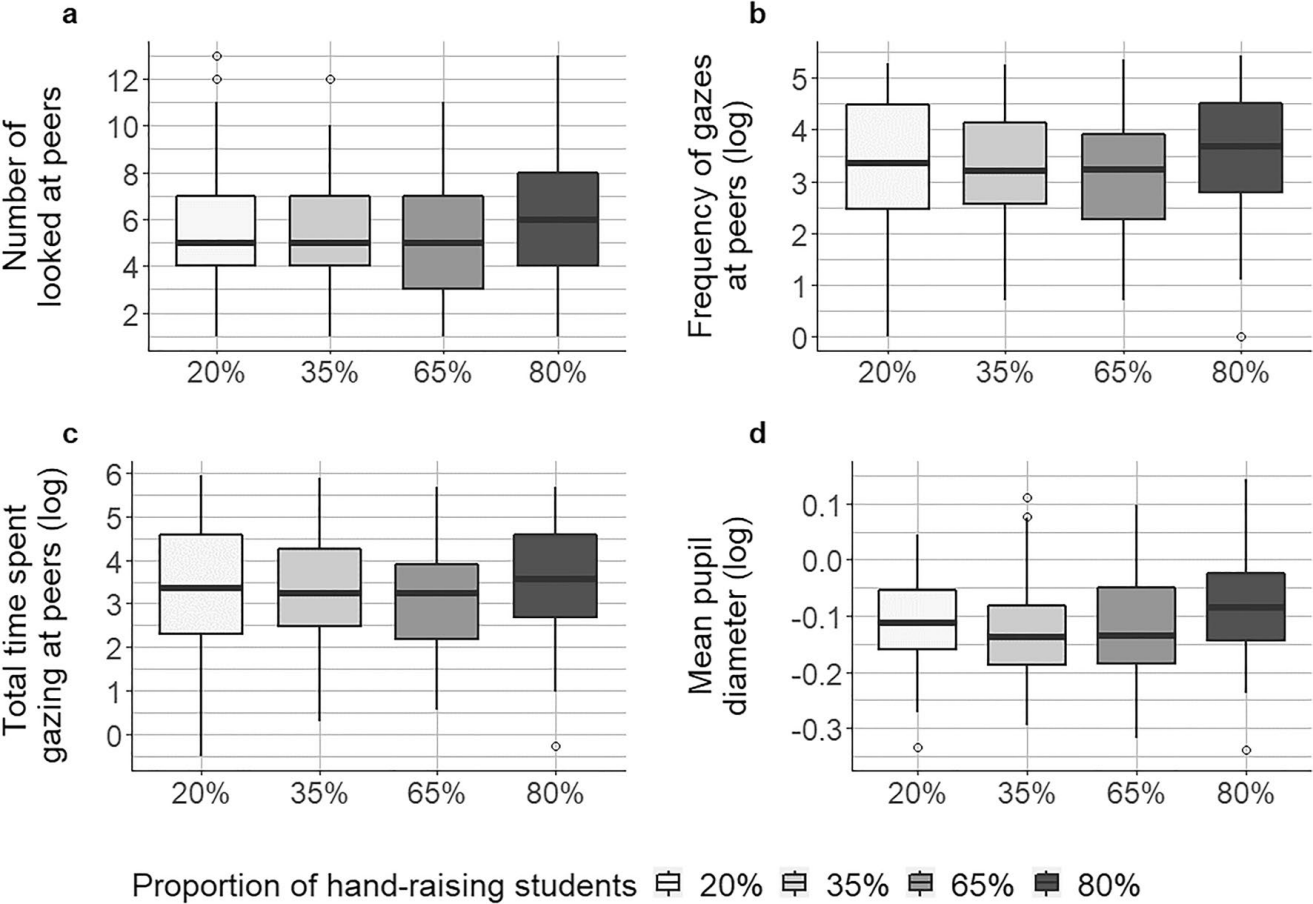
Boxplots for the eye-movement features in the different hand-raising conditions. The proportion of hand-raising students refers to the experimental variation of peer learners’ performance level via their hand-raising behaviour.

We found different results for the number of peer learners looked at compared to the frequency and total time they were looked at, indicating that these eye-movement features reflect different processing activities. With regard to the *number of peer learners looked at* (Fig. 2a; statistics for the full regression model in Supplementary Appendix 2), we found no significant difference between the 20% compared to 35% (*p* = 0.994) and 20% compared to 65% (*p* = 0.300) hand-raising conditions, but we found a significant difference between the 20% and 80% hand-raising conditions (*β* = 0.09, *SE* = 0.04, *t* = 2.06, *p* = 0.040, *d* = 0.20). Notably, the number of peer learners looked at was descriptively lower in the 65% hand-raising condition compared with the 20% condition, but significantly higher in the 80% condition (vs. 20%). By contrast, whereas the descriptive pattern of results was similar for the *frequency of gazing at peer learners* (Fig. 2b) and the *total gaze time on peer learners* (Fig. 2c), we found no statistically significant differences in how often and how long participants looked at the virtual peer learners in the different hand-raising conditions (*p-*values > 0.05; see statistics for the full regression models in Supplementary Appendix 2). These findings indicate that the hand-raising conditions affected the extent to which students noticed and actively attended to their peers’ behaviour (i.e., the *number of peers looked at*), but less so the intensity and time students spent processing the social comparison information (i.e., the *frequency of gazing at peer learners* and the *total gaze time on peer learners*).

### Students show greater pupil diameter for extreme hand-raising levels of peer learners, particularly when the clear majority is high-achieving

With regard to the effects on *mean pupil diameter* (Fig. 2d), students showed greater pupil diameter for the 20% and 80% hand-raising conditions, particularly when the clear majority of peer learners was high-achieving. The differences between the 20% and 35% hand-raising conditions as well as between 20 and 65% were not statistically significant (*p* = 0.433 and *p* = 0.328, respectively; see statistics for the full regression model in Supplementary Appendix 2). However, the 80% hand-raising condition led to a statistically significantly higher mean pupil diameter compared with the 20% hand-raising condition (*β* = 0.20, *SE* = 0.08, *t* = 2.45, *p* = 0.014, *d* = 0.52), suggesting that a majority of high-achieving peers (i.e., 80% of students raising their hands) led to a considerably higher level of mental effort and arousal for participants when processing this information compared to when a minority of peers exhibited high-achieving behaviour.

### Eye-tracking features as indicators of social comparison information show relations to situational self-concept

In addition to the effect of the experimental hand-raising conditions on students’ visual attention and pupil diameter, we examined how these behavioural indicators of active social comparisons relate to differences in students’ situational self-concept. In line with the theoretical assumptions underlying the BFLPE (i.e., social comparisons in the classroom lead to differences in individual academic self-concept), we expected students’ situational self-concept to be related to visual attention to peer learners (i.e., number of peers looked at, frequency of gazing at peers and total gaze time on peers) as well as to associated mental effort and arousal (indicated by the mean pupil diameter). The results revealed the expected relations for all three indicators of visual attention to peer learners: We found a statistically significant negative effect on students’ self-evaluations for the *number of peer learners looked at* (*β* = − 0.13, *SE* = 0.05, *t* = − 2.56, *p* = 0.010), the *frequency of gazing at peer learners* (*β* = − 0.11, *SE* = 0.05, *t* = − 2.06, *p* = 0.040), and the *total gaze time on peer learners* (*β* = − 0.10, *SE* = 0.05, *t* = − 2.27, *p* = 0.023). The *mean pupil diameter* was not related to differences in participants’ situational self-concept (*p* = 0.100). Detailed statistics for the full regression models are provided in Supplementary Appendix 3. These findings suggest that the active processing of social comparison information—indicated by visual attention to peer learners—was in fact related to students’ self-evaluations.

Lastly, we examined whether students’ eye movements can explain the impact of classmates' hand-raising behaviour on individual learners’ situational self-concepts. The results revealed that only contrasting the 20% and 80% hand-raising conditions against each other predicted students’ situational self-concept: In line with the BFLPE, the 80% hand-raising condition (i.e., higher-achieving peer learners) led to a statistically significantly lower situational self-concept than the 20% hand-raising condition (*β* = − 0.12, *SE* = 0.04, *t* = − 2.60, *p* = 0.009, *d* = 0.25). Notably, this predictive effect of the experimental hand-raising conditions on situational self-concept (i.e., the 80% hand-raising condition resulting in a statistically significantly lower self-concept compared to the 20% hand-raising condition) remained statistically significant in all three models when eye-movement features were additionally examined as predictors. Full statistics for the regression models are provided in Supplementary Appendix 3. Hence, our results indicate two types of effects on students’ self-concept: (a) a general psychological effect of classmates’ achievement-related behaviour in the classroom (i.e., the experimentally manipulated social comparison information affected students’ self-evaluation) and (b) a differential effect of interindividually different processing of social comparison information (i.e., students’ active social comparison behaviour—indicated by their visual attention to peer learners and mean pupil diameter—was related to their situational self-concept). We will discuss these findings in more detail below.

## Discussion

As most prominently demonstrated by BFLPE research, the consequences of social comparisons in real-world classrooms are well-known^4,8,11^. However, how exactly such social comparisons proceed—i.e., how students make use of social (comparison) information in the classroom—has so far remained unclear. To determine the extent to which students in fact actively engage in social comparisons with their peer learners, we used an IVR classroom as a standardised but authentic research setting with experimental variation in peer learners’ achievement-related behaviour (i.e., different proportions of peers who raised their hands). Moreover, we used eye-tracking data (i.e., students’ visual attention towards and pupillary response to their peers) to examine (1) the extent to which students attend to and respond to peer learners’ achievement-related behaviour, and (2) how students’ eye movements, as indicators of social comparison processes, are related to differences in situational self-concept. We found that

1. the different levels of hand-raising behaviour had an effect on students’ visual attention toward their virtual peer learners and their mean pupil diameter and that
2. markers of students’ visual attention toward their virtual peers were related to students’ situational self-concept.

**Figure 3:**
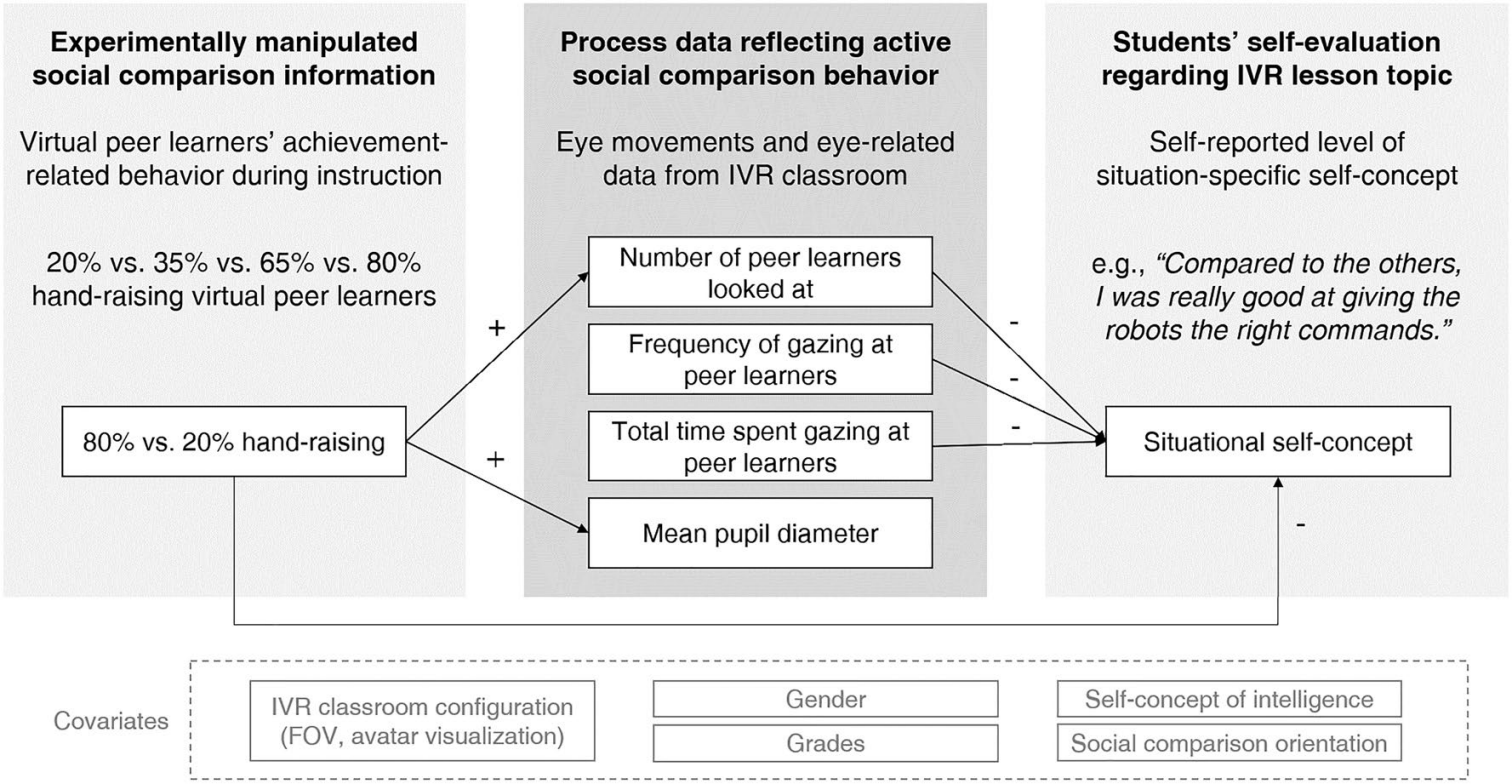
Overview of the revealed effects summarised in one structural model. A summary of all statistically significant relationships from the different statistical models is depicted. We included several covariates in the model to account for potential factors of influence in the social comparison context and in the specific IVR classroom configuration. Detailed information on the covariates is included in the Method section and in the Supplementary Information. FOV = field of view.

Figure 3 gives an overview of the results. Speaking to the results of our study, we found effects in line with our expectations (i.e., peers’ hand-raising affected the number of peer learners looked at and students’ mean pupil diameter), but at the same time, some of the relationships we expected turned out to be nonsignificant (i.e., the effect of the hand-raising conditions on how frequently learners gazed at and how long they spent gazing at peers). We would like to highlight that our study was the first to examine these relationships as such, and therefore, we also consider the lack of significant effects on some of the markers of visual attention to peers to be important results of our study. We argue that there is a good explanation for the pattern of results that we found, namely, that the markers of visual attention we examined and pupil diameter reflect different levels of engagement in social comparisons that are differentially influenced by situational and interindividual differences (i.e., a simple ‘noticing’ of social comparison information and the corresponding affective arousal in response to it vs. a deeper and possibly more intentional processing of this information). We discuss the results and our explanation for them in more detail in the following.

As with all behavioural indicators of cognitive processes, there are certain degrees of freedom involved in identifying observable behaviours that reflect internal processes^54^, such as social comparisons. We argue that an evaluation of what is—or is not—a ‘good’ behavioural indicator is ideally based on theoretical reasoning in combination with experimental studies that examine the relationship. To date, there is little to no research on specific behavioural indicators of social comparisons; therefore, we based our argumentation on the well-established concept of selective visual attention^55^, which provides a solid foundation from which to argue that the chosen features of students’ visual attention to virtual peer learners (i.e., the number of peer learners participants looked at, how frequently participants gazed at peers, and the total time spent gazing at them) are valid indicators of ongoing social comparison processes. Studies focussing on mutual gazing and cultural learning have shown that gaze direction is a key predictor of people’s actions or emotional states in social situations^56^. In fact, in the present study, selective visual attention to peers might be a particularly valid proxy for social comparisons, as all virtual classmates were unknown to the participating students, and observing the peers during the IVR lesson was the only way to get information about them. Yet, future studies should work toward providing additional evidence of this link (see future research perspectives on the basis of our findings in more detail below).

Importantly, when interpreting the results, one needs to bear in mind that all of the observed effects were relatively small. Far-reaching conclusions should therefore be drawn with caution. However, the a-priori power analysis we conducted for the small effects we expected determined that we had 90% power with the sample size we had. Therefore, when considering recent suggestions for interpreting effect sizes^57^, we consider the small effects we found to be meaningful, especially in light of the minimal intervention that led to them (only a 15-min exposure to a temporary event, i.e., different peers’ hand-raising behaviour). In fact, the small effect sizes obtained for the relationship between students’ visual attention to peers and their situational self-concept are not surprising when considered against the background that many intervention studies have reported small-sized effects on students’ self-concepts^58^. Moreover, we aggregated students’ visual attention to peers and their pupil diameters across the entire IVR lesson, including hand-raising situations as well as situations without any specific peer activity, whereby including situations without any specific peer activity might reduce the effects of the hand-raising. On a more general level, whereas the study clearly shows the potential of IVR technology in allowing novel insights and methodological approaches, the respective relationships and research designs have not explicitly been examined or employed in this manner before. Therefore, replications and cumulative support are needed.

Overall, our experimental findings provide important insights that help to answer the open question about students’ active social comparison behaviour in the classroom, specifically in an IVR setting. Whereas previous research has consistently highlighted the importance of social comparisons for students’ self-evaluations, it has not answered the question of whether students do in fact *actively* compare themselves with their peer learners or whether they are instead affected by comparisons based on grades or comments made by their peers or teachers. On the basis of behavioural indicators of social comparisons, our results indicate that participants actively attended and responded to their virtual peers' hand-raising behaviour in the IVR classroom. Moreover, our findings provide evidence of both general effects of the experimental conditions (i.e., peer learners’ achievement-related behaviour) and interindividual differences in social information processing.

Firstly, with regard to general psychological effects of the experimentally manipulated hand-raising behaviour of peer learners, the results indicate that students’ behavioural responses to their peer learners’ performance-related behaviour do not simply reflect the amount of activity happening in the classroom (i.e., more hand-raising) but rather the amount of social information that is processed. We found that visual attention to peer learners (i.e., how many different virtual classmates participants looked at as well as how often and for how long participants looked at virtual classmates in general) and mean pupil diameter were greatest in the 20% and 80% hand-raising conditions. More precisely, very low or very high performance by peer learners (i.e., a clear minority or majority raising their hands) seems to provide more social comparison information to students compared with more moderate levels of peers’ hand-raising (i.e., 35% and 65% of students raising their hands). This finding is in line with psychological research on social comparisons suggesting that contrastive social comparison effects (i.e., negative self-evaluations in response to a high-performing reference group) are more likely when the comparison information is more extreme and potentially unambiguous^59^. Taking a closer look at the 80% compared with the 20% condition, we did in fact find the expected pattern in which more hand-raising behaviour resulted in participants looking at a larger number of peer learners and larger pupil diameter. On the basis of these results, it can be argued that students notice and respond more to clearly interpretable social comparison information, which can in turn be considered a prerequisite for respective responses, reflected in effects on self-evaluations. It is thus not surprising that we found significant direct effects of the hand-raising conditions on students’ situational self-concept when comparing the conditions with a clear minority/majority of classmates raising their hands (i.e., 80% hand-raising peers led to significantly lower situational self-concept in students compared with 20% hand-raising peers).

Secondly, with regard to differential effects of social information processing, our results suggest that actively engaging in social comparisons can be observed at two levels that are differentially influenced by situational and interindividual differences. Whereas the *number of peer learners looked at* and *mean pupil diameter* differed significantly between the hand-raising conditions, the *frequency of gazing* and the *total time spent gazing at* peer learners were not affected by the proportion of hand-raising classmates. Notably, the number of peer learners looked at and mean pupil diameter are argued to reflect a simple ‘noticing’ of social comparison information and a corresponding affective arousal^38,60^ in response to social comparison information (especially in light of the fact that the content of the present IVR lesson was not particularly difficult for students; see details in the Method section). Noticing more peer learners and increased affective arousal might be due to the fact that the social comparison information that was provided (especially in the 20% and 80% hand-raising conditions) is particularly conspicuous. For instance, pupil diameter has repeatedly been found to be higher when people viewed emotionally pleasant or unpleasant information^34–37^, with particularly negative or threatening stimuli leading to increased pupillary responses^39,61,62^. In turn, the frequency and duration of gazes at peer learners indicate a deeper (and possibly more wilful) level of processing of this information^31,32^. Considering the effects of the different hand-raising conditions on the number of peers looked at and the mean pupil diameter but not the frequency of gazing or total gaze time, we argue that situation-specific social comparison information is noticed and responded to by students, but the extent to which students process this information seems to depend on factors that are unrelated to the situation (such individual competence beliefs or social orientation; see Supplementary Appendix 2).

With regards to effects on situational self-concept, the results revealed no relationship between students’ situational self-concept and their mean pupil diameter during instruction but significant relationships with all three indicators of visual attention to peer learners (i.e., number of peers looked at, frequency of gazing at peers, and total time spent gazing at peers); the higher the number of peers looked at and the more often or the longer students looked at their virtual classmates on average, the lower their situational self-concept. In other words, regardless of whether students’ visual attention was (at least partially) driven by their peers’ hand-raising behaviour, students who actively attended more to their peers’ performance gave themselves worse evaluations. We purposefully speak of a relationship between students’ visual attention on virtual peers and their self-concept as our analyses cannot establish perfect proof of a causal effect. However, we would like to highlight that the pattern of results was stable when including relevant controlling variables (e.g., students’ academic achievement, prior interest in the topic, and general self-concept of intelligence; see Supplementary Appendix 3), which supports the assumed direction of the effects as suggested by the BFLPE and associated social comparisons.

The present study varied virtual classmates’ hand-raising behaviour as an indicator of performance. Importantly, in previous research, students’ hand-raising has been found to be associated with achievement, but it has also been interpreted more generally to be an indicator of behavioural engagement^63^ and motivation^64^. In the present study, we made sure that the IVR lesson was easy to follow, whereas the teacher’s questions in the IVR lesson had a level of difficulty that was demanding for the participating students (as they referred to new content and required knowledge transfer and application)^65^ to ensure (a) that participating students perceived the virtual classmates as similar enough that it made sense to compare themselves with the virtual classmates^1,59^, and simultaneously, (b) that knowing the response to the teacher’s questions was still perceived as an indication of high performance. In this vein, we had the virtual classmates who raised their hands and were called on always give correct and thorough answers to the teacher's questions to emphasise that hand-raising was an indicator of performance. Whereas a related study in the IVR classroom provided evidence that the experimental manipulation of hand-raising was indeed associated with participants’ perceptions of the performance level of the class^66^, it is important to note that the manipulation of hand-raising behaviour presents only one naturally occurring piece of performance-related information that students can use for their social comparisons. Against this background, we consider our results to be particularly meaningful, as they indicate that students already attend and respond to more subtle performance-related behaviour (e.g., their peers’ hand-raising) when they make social comparisons.

The BFLPE has been found to be generalisable across countries with both more individualistic and more collectivistic cultures^8^. However, the way in which students see themselves in relation to others—and the way they might therefore interact with their peers and interpret their peers’ performance-related classroom behaviour—is culturally dependent^67^. In this vein, the extent to which hand-raising naturally occurs on the basis of students’ desires to contribute to the classroom discussion depends on the (cultural) dynamic in the classroom. For instance, in more collectivistic cultures, it might be normal for most classmates not to raise their hands if some students are already contributing to the discussion because they might not feel the need to do so (i.e., the lack of hand-raising would not be interpreted as an indication of low performance but would instead reflect the collectivist spirit). Against this background, we would like to highlight that the sample in the present study comprised sixth-grade students from Germany, which is commonly seen as an individualistic culture^68^. German students, particularly those attending so-called Gymnasium schools as our sample did (i.e., the highest track in the secondary school system), typically place great importance on their individual achievement in school. To ensure that hand-raising in our IVR classroom had meaning as performance-related behaviour, we designed the teacher-student interactions in our IVR lesson so that they would further emphasise the performance of individual students rather than the class as a whole; all the teacher’s questions were formulated in such a way that they addressed each individual student, and the teacher’s gaze was evenly distributed across the class to clearly show that each question was meant for every student.

Manipulating only one naturally occurring behavioural indicator of peers’ performance might be a reason that we found only small effects. At the same time, our manipulation of only one indicator calls for future research to investigate whether similar (or even more pronounced) effects occur with other or additional manipulations of classmates’ performance-related behaviour and—to extend the focus of the present study—manipulations of social comparison information beyond the information that can be gleaned from peer learners’ behaviour.

Turning to future research perspectives based on our findings, it is important to highlight that IVR environments—including standardised eye and gaze measures—provide a promising avenue for gaining insights into processes such as social comparisons in complex and dynamic environments. In the present study, we selected and aggregated four eye and gaze markers across the duration of the IVR lesson, thus allowing us to gain insights into the processing of social comparison information in a classroom (i.e., the number of peers looked at, how frequently learners gazed at their virtual peers, the total time spent gazing at virtual peers, and mean pupil diameter). We would like to highlight that we did not analyse students’ eye movements that were tied to shorter and specific time frames (e.g., those linked to peers’ hand-raising behaviour), and we did not assess students’ situational self-concept at multiple occasions during the IVR lesson to avoid disrupting students’ immersive experience in the IVR classroom. Whereas the IVR lesson lasted for only 15 min, and the posttest questionnaire was administered immediately afterwards, our study does not allow direct inferences to be drawn between students’ eye movements and effects of social comparisons. To further extend the insights gained from the present study, we suggest that future studies consider a more fine-grained analysis of gaze data as well as additional eye movement features and examine how these behavioural indicators develop over time. For instance, it would be interesting to see whether there are certain peer learners that students’ visual attention keeps returning to or whether students follow the teacher’s gaze and increasingly look at (and consequently compare themselves with) the students that are the teacher’s focus of attention. In addition, the use of continuous time models to examine the interrelationships and potential reciprocal dependencies of different types of measures (e.g., changes in pupil diameter as a reaction to visual attention to peers) and the development of certain behaviours over time (e.g., overall declining or increasing visual attention to peer learners or a focus on peer learners, particularly while they are raising their hands).

Notably, we focus our discussion on the results that are directly related to our research questions. With regard to our study design, we varied not only peers’ hand-raising behaviour but also the location of participating students’ seats in the classroom and the avatars’ style. The question of exactly how the configuration of the IVR classroom affects how students learn, and more specifically, how students perceive the IVR lesson and their virtual classmates is the focus of another IVR classroom study^69^ that found that the IVR configuration features affect how students distribute their visual attention during instruction and identified relationships to students' interest and learning in the IVR classroom. In the present study, we controlled for potential confounding effects of the additional IVR classroom configurations by including them as covariates in all the analyses. Importantly, the IVR conditions did not affect the results of the regression models used to predict students’ situational self-concept. However, despite the fact that we statistically controlled for confounding effects, we cannot fully exclude the possibility that the additional IVR configurations distorted the results reported in this paper to some extent. One could argue that the results of this study would likely be more pronounced in a research design that did not include the additional IVR conditions, but more studies will need to be conducted to provide the respective evidence.

To successfully use IVR as a tool for classroom research, it is crucial to design authentic IVR classroom experiences that make students react as they would in the real world^70^. Unfortunately, it is difficult to empirically evaluate the authenticity of IVR classrooms: Ideally, IVR settings such as the one used in the present study could be compared with real-world settings to establish evidence of their authenticity; however, such evidence is difficult to achieve when considering the uncontrollable nature of real-world classrooms. We used recordings and motion captures that stemmed from a real sixth-grade classroom to ensure that our IVR simulation would reflect an authentic classroom experience for the participating sixth graders. Importantly, what we refer to as an ‘authentic’ IVR experience can be described on a number of different dimensions (e.g., presence, realism, immersion), but no unified definitions of any of them can be found in the existing literature to date; moreover, considering the fast-paced technical advances in the field of software and hardware development, existing conceptualisations of IVR experiences are quickly missing the latest state of modern IVR technology^49,50,71–73^. Given the limitations involved in empirically assessing the authenticity of IVR environments, specifically IVR classrooms, we consider the issue of authenticity to instead be an issue of validity. To this end, there are empirical findings that support the idea that IVR can provide an authentic and valid research environment. For instance, there is evidence that students’ individually different reactions to distractions in real-world classrooms are similar in an IVR classroom, and the respective differences (e.g., associated with ADHD diagnoses) can be reproduced in an IVR classroom simulation^45,74,75^. More generally speaking, children have been found to be particularly (both cognitively and behaviourally) responsive to IVR environments and tend to perceive the simulations as more real and feel a higher level of presence, which makes them act more spontaneously while thinking less about the world outside of the IVR environment^48,76,77^. In our study, we assessed the level of presence that participants experienced in the IVR classroom on the basis of common conceptualisations of spatial and social presence, and we asked participants to rate the degree of realism of the IVR lesson. As students’ self-reports (see details in Supplementary Appendix 1) indicated, they perceived the IVR lesson, including the events and people in it, as rather realistic and similar to what they would experience in real-world classrooms. Notably, as the authors of a recent systematic review of IVR applications in higher education^72^ pointed out, almost all IVR studies claim to have created ‘realistic environments.’ However, there are large differences with regard to what is understood as ‘realistic’ in each of the studies. In locating the present study on the scale that the review outlined, we find ourselves at the high-end of ‘VR environments with complex, high-quality graphics’ that allow for a ‘high-fidelity’ IVR experience^72^. At the same time, when interpreting the results of an IVR study such as the present one, one needs to acknowledge that—no matter how realistic it is—the IVR classroom is a simulation and is naturally not the same as the real-world classroom environment that participants are used to (i.e., so-called ‘dual reality’^73^). Nevertheless, given that the experiment has been carefully designed and considers central guidelines for immersive VR research^73^, the present IVR study made it possible to use the affordances of IVR technology to address a central shortcoming of experimental research on social comparisons to date—that the respective measures have relied on ‘detached classroom and experimental situations’^78^. With the IVR classroom simulation, the present study provided a more naturalistic and yet standardised experimental setting for the measures associated with social comparisons compared with traditional experimental set-ups. Moreover, the IVR classroom made it possible to objectively trace students’ behavioural responses to social comparison information in the classroom, specifically to assess participants’ visual attention and pupillometry nonintrusively and under standardised (lighting) conditions, which allowed us to use these measures in a valid way in accordance with the sociophysiological literature. Ultimately, the validity of IVR environments must be fully established through a series of empirical results that are in line with existing theories and must reproduce real-life human behaviours in the IVR, such as the replication of the classic Milgram experiment^47^ or the replication of social facilitation and inhibition effects^79,80^. We argue that our study presents an important step in this direction.

In conclusion, we were able to extend existing research on the BFLPE and provide experimental support for the role of active social comparisons during instruction by using eye-movement data from an IVR classroom. In line with the claim that new technologies allow researchers to bridge the gap between ‘experimental and methodological rigorousness on the one hand, and the complexity and uncontrollable nature of an authentic classroom full of pupils’^81^, we see IVR as a tool that will allow researchers to further advance research on social comparisons and similar phenomena in classrooms and beyond. We believe that the approach presented in this study provides an important foundation for future work that will extend the present insights and apply this approach to other topics as well.

## Methods

This research complies with ethical standards of research with human subjects, confirmed by the ethics committee at the University of Tübingen, Faculty of Economics and Social Sciences (date of approval: November 25, 2019, file number: A2.5.4-106_aa). In addition, regional educational authorities approved the study and the data collection, and all research was performed in accordance with relevant guidelines and regulations. We obtained written informed consent from both the participating students and their parents or legal guardians prior to students’ participation in the study.

### Participants

We recruited *N* = 381 students in Grade 6 from local academic-track schools via e-mails and invitation letters. To determine the required sample size, we computed an a-priori power analysis considering existing findings from experimental studies^82,83^. Since we expected our manipulation to be less salient and effects on behavioural responses less powerful than in these studies, we assumed small to medium effects (*f* = 0.20). Based on this, a necessary sample size of *n* = 90 students in each of the four hand-raising conditions was determined for the respective analyses of variance (for two-tailed tests with a 0.05 alpha level and a minimum power of 0.90). Due to technical issues during data collection (i.e., visual or audio issues with the HMDs during the IVR experience), data from 28 participants had to be excluded from the analyses. The cleaned sample consisted of *N* = 353 students (*M*_*Age*_ = 11.52 years, *SD*_*Age*_ = 0.55; 46.7% girls).

### Content and course of the IVR lesson

The IVR lesson’s content was adapted from tested and evaluated materials from a course designed to teach kids basic computational thinking skills^84^. The students learned about the meaning of coding and sequences and loops as basic computational concepts and worked on two exercises. The students’ self-reports indicated that they found the lesson easy to follow (perceived difficulty assessed with 10 items on a 4-point rating scale, with higher values indicating higher difficulty, yielded a mean value of* M* = 1.38, *SD* = 0.42; Cronbach’s alpha 0.86). As it is not commonly included in the curricula of primary or lower secondary schools, the lesson topic was chosen so that social comparisons could be investigated in an unbiased context largely independently of any previous experience in this subject.

The entire IVR lesson lasted about 15 min and took place in a simulated classroom showing a typical teaching situation with explanations by the teacher, dialogue between the teacher and the virtual students, and independent work on exercises. We used audio recordings and motion captures stemming from a real classroom to ensure that the pace and content of the virtual students’ answers and their movements were calibrated to be typical of sixth graders. The IVR experience was designed and rendered using the Unreal Game Engine v4.23.1. Figure 1 shows the design of the virtual classroom.

### Configuration and design of the IVR classroom

We systematically varied the performance level of the IVR class and therefore manipulated the virtual classmates’ hand-raising behaviour (i.e., the number of students raising their hands in response to the teacher’s questions or indicating that they knew the correct solution to a task). The virtual classmates’ hand-raising behaviour was manipulated on four levels, with 20% vs. 35% vs. 65% vs. 80% of students raising their hands and showing high-performing participation. We chose these four experimental hand-raising conditions to obtain the best possible balance between three aspects: (a) an effective study design, (b) a realistic representation of hand-raising behaviour in a classroom, and (c) a differentiated picture of when aversive versus positive effects appear. Hence, we (a) limited our study design to four hand-raising conditions to have enough participants per group to obtain sufficient statistical power and chose four experimental conditions that were comparable and allowed the exact same IVR lesson to take place (i.e., all conditions had students raise their hands to answer the teacher’s questions and the teacher called on one of them). Moreover, we (b) omitted the 0% and 100% hand-raising conditions from our experimental design, as these conditions were not likely to consistently appear for every question asked by the teacher during a lesson and would therefore be likely to lead to lower levels of perceived realism among the students participating in the IVR classroom. Lastly, we (c) decided to use relatively fine-grained differences between 20% and 35% and between 65% and 80%, whereas there was a larger difference between 35% and 65% to ensure differentiated grading and yet unambiguous information about whether the percentage of classmates who were high-achieving was below versus above average. Participants were randomly assigned to one of the hand-raising conditions and reported similarly high levels of experienced presence (following common conceptualizations of spatial and social presence^49,50^) and perceived realism of the IVR lesson (e.g., ‘What I experienced in the virtual classroom could also happen in a real classroom’) across all conditions (see details and respective statistics in Supplementary Appendix 1).

Notably, aside from peers’ hand-raising, we also manipulated other features of the IVR classroom. We varied participating students’ field of view (by seating them at either the front or the back of the classroom) and the avatars’ style. However, these variations were not relevant to the present study and are examined in a separate study that is focused on the features of IVR classrooms^69^. We chose this research design for its efficiency and economy and because it would help us attain the best possible outcome when considering (a) the necessary effort and resources involved in an IVR eye-tracking research project with a large sample of sixth-grade students and (b) the fact that research has not yet provided clear answers with respect to how an IVR classroom should be programmed. This design also provided ideal conditions as an experimental tool and enabled us to account for factors that might influence how the central information provided in the IVR classroom is perceived. With regard to the present study, we controlled for potential confounding effects of the additional IVR configurations by including them as covariates in all the analyses. Detailed information about the additional IVR configuration features is included in Supplementary Appendix 1.

### Experiment procedure

The experiment took place in a quiet room at the participants’ school and students participated in groups of up to 10. Before the beginning of each test session, head-mounted displays (HMDs) were set up for each participant. We used the HTC Vive Pro Eye HMD in our experiments. The researchers randomly assigned one of the experimental conditions to each set-up HMD by means of random number generation. Students were then allowed to enter the testing room and were free to choose any seat without knowing the experimental conditions (they were debriefed in detail after they had completed the experiment). All testing sessions followed the same procedure, and the experimental conditions differed only with regard to specific manipulations in the IVR classroom scenario that participants experienced.

First, participants filled out the first part of a paper-based questionnaire that included demographics, basic personality characteristics, and learning background (i.e., prior experience with the lesson topic and IVR). Second, participants put on the HMDs and were helped to calibrate the included eye trackers. Upon successful calibration of the eye trackers, participants experienced the IVR lesson (which lasted about 15 min). Participants all began the IVR lesson at the same time and were instructed to behave as they would in a normal classroom situation (e.g., look around, raise their hands) while remaining seated and quiet. Third, as soon as the participants finished the IVR lesson, they completed the second part of the questionnaire (including measures of self-concepts, experienced presence in the IVR, and perceived realism of the IVR classroom), followed by a debriefing. In total, each test session took approximately 45 min, including all instructions and preparation, and was supervised by research assistants throughout.

### Eye-tracking measures and data pre-processing

To collect eye movement data, we used the Tobii eye tracker integrated into the HTC Vive Pro Eye head-mounted display (HMD). The HMD has a refresh rate of 90 Hz and field of view of 110° (screen resolution 1440 × 1600), and the integrated Tobii eye tracker runs at a 120 Hz sampling rate. Before the start of the IVR lesson, we calibrated the eye tracker based on a 5-point calibration for each participant. During the experiments, continuous measures of HMD orientation, gaze, and eye-related data were collected (assigned to participants via an anonymous identifier) and aggregated as features for later analysis. A correlation matrix for the eye-movement features is provided in Supplementary Appendix 5.

### Pupil diameter

Pupil diameter was recorded in millimetres on a millisecond basis. In the course of data pre-processing, we smoothed and normalised the pupil diameter measures using the Savitzky–Golay filter^85^ and divisive baseline correction with a baseline duration of approximately 1 s from an interval at the beginning of the experiment^86^. For the purpose of the present study, we averaged the measure across the whole IVR experience and used the mean pupil diameter as an indication of participants’ arousal and mental effort^87^. The normalised mean pupil diameter ranged between 0.71 and 1.97 (*M* = 0.92, *SD* = 0.14). Because the mean pupil diameter was nonnormally distributed (skewness of 3.43, *SE* = 0.15; kurtosis of 19.95, *SE* = 0.29), we log-transformed the variable for the analyses.

### Visual attention at peer learners

With regards to participants’ visual attention, we defined virtual peer learners as the objects of interest (OOIs). We first applied a linear polynomial interpolation of degree one to clean the gaze data and account for missing values. Using head pose and gaze data, we then applied ray-casting^88,89^ to map the gaze into the 3D virtual environment. Calculating the intersections between predefined colliders of the OOIs with the gaze vectors allowed us to identify when participants looked at the OOIs (a detailed description of the ray-casting technique used to identify objects of gaze in the IVR can be found in Supplementary Appendix 4). Considering that objects of gaze may not directly represent visual attention, as participants can unconsciously gaze at an OOI for a very short time when looking around, we set an attention threshold of at least 500 ms to count OOIs. We obtained similar trends across different thresholds tested and chose the selected threshold as a conservative estimate that is larger than classical fixation thresholds applied for both conventional^90^ or IVR eye-tracking^91^ setups. We used the resulting information about the object of gaze to calculate the following variables:

- *Number of peer learners looked at:* We counted each peer learner that participants looked at for a minimum attention threshold of 500 ms, regardless of how often participants’ gaze rested on the respective OOI in total. There were a total of 24 peer learners in the IVR classroom; the actual number of peers looked at ranged from 1 to 13 (*M* = 5.58, *SD* = 2.76).
- *Frequency of gazing at peer learners:* We summed up the number of gaze shifts toward virtual classmates across the entire IVR experiment. We counted a new ‘gaze at peer learner’ as soon as the gaze shifted to a virtual peer learner from any other object for at least the attention threshold of 500 ms. The frequency of gazing at peer learners ranged from 1 to 230 (*M* = 47.63, *SD* = 46.45). The frequency of gazing at peers was nonnormally distributed (skewness of 1.41, *SE* = 0.15; kurtosis of 1.66, *SE* = 0.29); thus, we used a log transformation of the variable for the analyses.
- *Total time spent gazing at peer learners:* We summed up the duration of all intervals longer than the attention threshold of 500 ms which participants spent looking at a peer learner across the whole VR experiment. The total time spent gazing at virtual peer learners ranged from 0.60 to 382.49 s (*M* = 56.10, *SD* = 65.11). Time spent gazing at virtual peer learners was nonnormally distributed (skewness of 2.03, *SE* = 0.15; kurtosis of 4.94, *SE* = 0.29); thus, we log transformed the variable for the analyses.

### Self-report measures of students’ self-concept

We assessed participants’ situational self-concept after the IVR lesson with respect to the specific experience with virtual classmates in the IVR classroom. The self-concept scale consisted of four items (1. ‘I could not solve the robot tasks as easily as the other students in the virtual classroom’; 2. ‘Compared with the others, I was really good at giving the robots the right commands’; 3. ‘I could solve the robot tasks faster than the others’; 4. ‘It was harder for me to understand the robot tasks than for the other students’). Two of the items were reverse-scored and recoded accordingly. The four items were based on the Self Description Questionnaire (SDQ) III^92^ and thus used the wordings that are commonly found in self-reports of academic self-evaluations. Specifically, we used the validated German versions of the items^93^ and adapted them to target the specific domain and topic of the IVR situation (i.e., we replaced the subject in the original version of the items with the specific task in the IVR classroom, i.e., understanding and solving the robot task/giving the robots the right commands). A 4-point rating scale ranging from 1 (*not true at all*) to 4 (*absolutely true*) was applied; the scale had an acceptable Cronbach’s α of 0.71.

### Covariates

The BFLPE has been argued to generalise across diverse student characteristics and contexts^11,94^. However, specifically gender^95,96^, individual achievement^22,24^, and social orientation^19,97^ have repeatedly been discussed as factors of influence in the context of social comparison effects. To account for potentially relevant covariates in the social comparison context, we included participants’ gender, a proxy for their individual achievement in the IVR lesson, and their social orientation as covariates in our analyses to account for their potential confounding effects on students’ social comparison behaviour and situational self-concepts in the IVR classroom. As a proxy for individual achievement in the IVR lesson, we used students’ latest grades in mathematics and the German language from their school report cards and assessed participants’ prior interest in the topic and their self-concept of intelligence in the paper-based pretest questionnaire. Prior interest in the topic of computational thinking was assessed with five items (e.g., ‘I would like to know more about how computer programs or robots work’). Self-concept of intelligence was measured with four items (e.g., ‘I often think I'm not as smart as the others’)^93^. Social orientation was assessed with seven items (e.g., ‘I pay close attention to how I do things compared with my classmates’)^97^. All three scales were rated on a 4-point rating scale ranging from 1 (*not true at all*) to 4 (*absolutely true*) and had acceptable Cronbach’s α values of 0.91, 0.72 and 0.74, respectively.

### Regression analyses

Using the processed and accumulated eye-tracking data, we calculated multiple regression analyses to examine to what extent (1) the experimental variation of virtual peer learners’ hand-raising behaviour affected students’ pupillary response to and visual attention towards the social comparison information as well as (2) how these behavioural responses to the provided social comparison information were related to students’ situational self-concept. We calculated separate models for each of the outcome variables. Prior to the analyses, all continuous independent and dependent variables were z-standardised, and categorical variables were dummy-coded. We included the experimental hand-raising conditions as dummy-coded variables in the regression models and set 20% hand-raising classmates as the reference category to test the proposed relationship (i.e., increasing visual attention to peers and increasing pupil diameters with increasing proportions of hand-raising peers). The regression analyses were chosen to test our assumptions in the most efficient way. Along with the descriptive statistics reported in the paper, the regressions provide the central insights necessary to address the research questions. To account for the fact that each testing groups consisted of students within the same school, we controlled for cluster effects by using a school variable in all analyses (number of clusters *N* = 12).

Following the suggestion that potential confounding variables should also be considered in strictly randomised research designs^98^, we added relevant background variables to the regression models. The results on the effects we examined for both research questions remained the same when we included the covariates. To avoid presenting two sets of results that are very similar, and to provide the more comprehensive picture, the paper reports only the results from the regression models that included the covariates. The paper focusses on the main effects that address the research questions; detailed information about all of the covariates is included in Supplementary Appendices 2–3. Additional results of all analyses without covariates and only the set of covariates related to the experimental design of our study (i.e., students’ seating position and the style of the avatars in the IVR classroom) can be found in Supplementary Appendices 6–7.

All models were calculated in Mplus 8.2, using full information maximum likelihood estimation for missing values^99^. As we report standardised regression coefficients, these can be interpreted as effect sizes. We additionally calculated Cohen’s *d* for standardised mean differences of dummy-coded categorical variables, whereby values < 0.20 indicate small, values < 0.50 medium-sized, and values > 0.80 large effects^100^. Hypotheses were tested with two-tailed tests with a critical *p*-value and confidence intervals set at an alpha level of 0.05.

### Supplementary Information

Supplementary Information.

## Data Availability

We provide access to all data and data analysis scripts including the data pre-processing steps on the Open Science Framework (OSF) under the following link: https://doi.org/10.17605/OSF.IO/JB8VQ.
