# Supplementary material for: Investigating social comparison behaviour in an immersive virtual reality classroom based on eye-movement data
Source: Sci Rep. 2023 Sep 6;13:14672. doi: 10.1038/s41598-023-41704-2 (PMC10483041; doi:10.1038/s41598-023-41704-2)
Supplement: Supplementary file 1 — Supplementary Information. [file 41598_2023_41704_MOESM1_ESM.pdf]

Investigating Social Comparison Behaviour in an Immersive Virtual Reality Classroom Based on Eye-Movement Data

Lisa Hasenbein[1,*], Philipp Stark[1], Ulrich Trautwein[1], Hong Gao[2], Enkelejda Kasneci[2], and Richard Göllner[1,*]

[1] Hector Research Institute of Education Sciences and Psychology, University of Tübingen, Europastraße 6, 72072 Tübingen, Germany
[2] Human-Centered Technologies for Learning, Technical University of Munich, Arcisstraße 21, 80333 Munich, Germany
[*] lisa.hasenbein@uni-tuebingen.de, richard.goellner@uni-tuebingen.de

SUPPLEMENTARY INFORMATION

Supplementary Appendix 1.	Details on the configuration of the IVR classroom and students' experienced presence and perceived realism
Supplementary Appendix 2.	Effects of virtual peers' hand raising on students' eye movements
Supplementary Appendix 3.	Relationship between students' eye movements and their situational self-concept
Supplementary Appendix 4.	Details on the ray-casting technique used to identify objects of gaze in the IVR
Supplementary Appendix 5.	Correlation matrix for the eye-movement features
Supplementary Appendix 6.	Additional analyses: Effects of virtual peers' hand raising on students' eye movements (without covariates)
Supplementary Appendix 7.	Additional analyses: Relationship between students' eye movements and their situational self-concept (without covariates)

We provide access to all data and data analysis scripts including the data pre-processing steps on the Open Science Framework (OSF) under the following link: https://doi.org/10.17605/OSF.IO/JB8VQ.

Supplementary Appendix 1

Details on the configuration of the IVR classroom and students' experienced presence and perceived realism

As research has not yet provided clear answers as to how an IVR classroom should be programmed to provide ideal conditions as an experimental tool, our experimental design included systematic variations of (a) participants' seating position in the IVR classroom and (b) the virtual avatars representing the peer learners and the teacher. We added these configuration conditions in addition to the four hand-raising conditions to make sure we accounted for factors that might influence how the social information we provided in the IVR classroom was perceived. More specifically, we varied participating students' seating position on two levels and allocated them to either the front (i.e., second of four rows) or the back (i.e., fourth of four rows) of the IVR classroom. The virtual classmates' and teacher's avatars were varied on two levels as well and were designed to be either more cartoon-like or more realistic. Participants were randomly assigned to one of the conditions, and their allocation to the different seating positions and avatar representations was counterbalanced with respect to the main experimental conditions with different percentages of hand-raising peers.

Importantly, being seated in the front meant that students were seated in the second (of four) rows of benches in the classroom. Looking toward the front of the classroom and at the teacher from this position, students could still see at least 7 virtual classmates. Importantly, we distributed the proportion of hand-raising peers equally across Rows 1-2 and 3-4 to ensure that the participating students had the same comparison group regardless of the position of their seat in the front/back of the classroom, particularly when they were seated in the front (a) when they turned around and looked at the whole class as well as (b) when they did not turn around and barely looked at the last two rows.

We checked for participants' perception of an authentic IVR classroom experience via self-reports. Therefore, we assessed participants' level of experienced presence in the IVR classroom with nine items (e.g., "I felt like I was sitting in the virtual classroom" or "I felt like the teacher in the virtual classroom really addressed me") based on common conceptualizations of spatial and social presence[1,2]. Moreover, we asked participants to rate the degree of realism of the IVR lesson with six items (e.g., "What I experienced in the virtual classroom could also happen in a real classroom"). Both variables were rated on a 4-point rating scale ranging from 1 (*not true at all*) to 4 (*absolutely true*) and had acceptable Cronbach's alpha values of .77 and .78, respectively. The self-reports for experienced presence and perceived realism indicated high levels of experienced presence and perceived realism in the IVR environment across all configuration conditions: The reported mean levels of experienced presence and perceived realism ranged from 2.82 to 2.97 ($0.52 < SD$s < 0.62) in all configuration conditions. None of the configuration conditions had a statistically significant effect on participants' experienced level of presence or perceived realism (all p-values > 0.05).

Table S1 below provides descriptive statistics for all outcome variables in the different hand-raising conditions (comparable to Table 1 in the paper), differentiating between the front and back seating positions. Importantly, descriptive statistics for all our outcome variables show a similar pattern across the hand-raising conditions in both the front and back seating positions, and this pattern reflects the descriptive statistics for the full sample (as they are reported in the paper). When conducting the regression analyses separately for the seating positions, we obtained similar effects for the hand-raising conditions on all the eye-movement features we examined; however, they did not reach statistical significance, as the sample size was much smaller.

Experimental condition			Number of peers looked at	Frequency of gazing at peers (log)	Total time gazing at peers (log)	Mean pupil diameter (log)	Situational self-concept
		N	*M (SD)*	*M (SD)*	*M (SD)*	*M (SD)*	*M (SD)*
Seating position: Front	20% hand-raising	38	4.32 (1.76)	2.57 (1.03)	2.49 (1.19)	-0.11 (0.08)	3.53 (0.45)
	35% hand-raising	38	4.74 (2.44)	2.76 (0.90)	2.71 (0.96)	-0.10 (0.15)	3.48 (0.57)
	65% hand-raising	39	4.39 (2.49)	2.65 (0.90)	2.59 (0.84)	-0.08 (0.16)	3.46 (0.44)
	80% hand-raising	36	4.59 (2.51)	2.65 (0.89)	2.58 (0.93)	-0.05 (0.12)	3.45 (0.46)
Seating position: Back	20% hand-raising	54	6.49 (3.11)	3.99 (0.90)	4.11 (1.00)	-0.11 (0.08)	3.41 (0.58)
	35% hand-raising	48	6.18 (3.12)	3.67 (1.20)	3.81 (1.35)	-0.13 (0.09)	3.41 (0.49)
	65% hand-raising	46	5.76 (2.67)	3.65 (1.13)	3.62 (1.26)	-0.11 (0.11)	3.36 (0.50)
	80% hand-raising	54	7.00 (2.30)	4.06 (0.84)	4.16 (0.94)	-0.05 (0.16)	3.23 (0.68)

Table S1. Descriptive statistics for eye-movement features in different hand-raising conditions. Hand-raising refers to the experimental manipulation of peer learners' performance level via the proportion of hand-raising students.

References

1. Lombard, M., Ditton, T. B. & Weinstein, L. Measuring telepresence: The temple presence inventory in *12th Annual International Workshop on Presence* 1–15 (International Society for Presence Research, 2009).
2. Schubert, T., Friedmann, F. & Regenbrecht, H. The experience of presence: Factor analytic insights. *Presence* **10**, 266–281 (2001).

Supplementary Appendix 2

Effects of virtual peers' hand raising on students' eye movements

In the following, detailed statistics and standardized regression coefficients for the effects of the experimental hand-raising conditions on the different eye-movement features (i.e., the number of peer learners looked at, the frequency of gazing at peer learners, the total time spent gazing at peer learners, and the mean pupil diameter) are provided. As additional results, Supplementary Appendix 6 provides statistics for the regression models without the full set of covariates.

Effects on the number of peer learners looked at

Table S2 below provides detailed statistics and standardized regression coefficients for the effects of the experimental hand-raising conditions on the number of peer learners looked at.

Variables	β	*SE*	*t*	*p*-value	95% CI
(Intercept)	-0.02	0.07	-0.31	0.758	[-0.17, 0.12]
35% vs. 20% hand-raising	0.00	0.07	0.01	0.994	[-0.13, 0.13]
65% vs. 20% hand-raising	-0.08	0.07	-1.04	0.300	[-0.22, 0.07]
80% vs. 20% hand-raising	0.09	0.04	2.06	0.040	[0.00, 0.17]
Back vs. front sitting position	0.29	0.06	5.21	<0.001	[0.18, 0.40]
Realistic vs. cartoon avatars	-0.24	0.07	-3.51	<0.001	[-0.38, -0.11]
Gender (male vs. female)	-0.11	0.07	-1.50	0.133	[-0.25, 0.03]
Math grade	-0.04	0.06	-0.68	0.494	[-0.16, 0.08]
German grade	0.16	0.07	2.45	0.014	[0.03, 0.29]
Prior CT interest	-0.09	0.06	-1.51	0.132	[-0.21, 0.03]
Self-concept of intelligence	0.09	0.04	1.96	0.050	[0.00, 0.17]
Social orientation	0.05	0.07	0.69	0.493	[-0.09, 0.19]

Table S2. Standardized regression coefficients predicting the number of peer learners looked at by the experimental hand-raising conditions. *SE* = Standard error; CI = Confidence interval. *p*-values refer to the standardized regression coefficients β. For categorical variables, the second category mentioned serves as the reference category. Grades were on a scale from 1 to 6 with higher numbers indicating lower achievement. Prior CT (Computational Thinking) interest, self-concept of intelligence, and social orientation were assessed on a 4-point rating scale with higher values indicating higher levels of these constructs.

With regard to the covariates, the results indicated that students' German grade and self-concept of intelligence had significant effects on the number of students they looked at: Students with worse German grades and higher self-concept of intelligence looked at more of their virtual peer learners, indicating that students' level of (domain-specific) confidence in their own abilities might have affected how often they turned their attention toward their peers. Moreover, with regard to the IVR configuration, the results revealed that the number of peer learners participants looked at was statistically significantly higher for participants who were seated in the back of the IVR classroom (d = 0.75), suggesting that the number of peer learners participants looked at increases when there are more peer learners in the participant's field of view. Moreover, the number of peer learners that participants looked at was significantly higher when the virtual classmates were presented as cartoon-style avatars (d = 0.57), indicating that students were generally more engaged with virtual peer learners when the peers were presented in a cartoon-style manner.

Effects on the frequency of gazing at peer learners

Table S3 below provides detailed statistics and standardized regression coefficients for the effects of the experimental hand-raising conditions on the frequency of gazing at peer learners.

With regard to the covariates, we found a positive effect of students' general self-concept of intelligence on the total time spent gazing at peer learners, suggesting that, whereas peer learners' achievement-related behavior (i.e., the hand-raising conditions) did not affect how long students looked at their peers, students who felt more comfortable about their intellectual abilities paid more attention to their virtual classmates. Similar to the number of peer learners participants looked at (see Table S1), the results regarding the IVR configuration revealed that the frequency of gazing at peer learners was statistically significantly higher for participants who were seated in the back of the IVR classroom (d = 1.24), suggesting that visual attention to peer learners increases when more peer learners are in the participant's field of view. The frequency of gazing at peer learners was also statistically significantly higher when the virtual classmates were presented as cartoon-style avatars (d = 0.80), indicating that students spent (or needed to spend) more time processing the social information provided by the peer learners' avatars when the avatars were less realistic.

Variables	β	*SE*	*t*	*p*-value	95% CI
(Intercept)	-0.01	0.06	-0.24	0.808	[-0.17, 0.10]
35% vs. 20% hand-raising	-0.03	0.03	-0.78	0.434	[-0.09, 0.04]
65% vs. 20% hand-raising	-0.09	0.08	-1.08	0.279	[-0.24, 0.07]
80% vs. 20% hand-raising	0.05	0.03	1.91	0.056	[0.00, 0.11]
Back vs. front sitting position	0.47	0.05	10.00	<0.001	[0.37, 0.56]
Realistic vs. cartoon avatars	-0.29	0.03	-11.78	<0.001	[-0.34, -0.24]
Gender (male vs. female)	-0.02	0.06	-0.34	0.737	[-0.14, 0.10]
Math grade	-0.01	0.06	-0.09	0.932	[-0.12, 0.11]
German grade	0.10	0.08	1.30	0.193	[-0.05, 0.25]
Prior CT interest	-0.10	0.07	-1.43	0.153	[-0.24, 0.04]
Self-concept of intelligence	0.13	0.04	3.50	<0.001	[0.06, 0.20]
Social orientation	0.09	0.05	1.94	0.052	[0.00, 0.18]

Table S3. Standardized regression coefficients predicting students' frequency of gazing at peer learners by the experimental hand-raising conditions. *SE* = Standard error; CI = Confidence interval. *p*-values refer to the standardized regression coefficients β. For categorical variables, the second category mentioned serves as the reference category. Grades were on a scale from 1 to 6 with higher numbers indicating lower achievement. Prior CT (Computational Thinking) interest, self-concept of intelligence, and social orientation were assessed on a 4-point rating scale with higher values indicating higher levels of these constructs.

Effects on the total time spent gazing at peer learners

Similar to the model predicting the frequency of gazing at peers (see Table S3), we found a positive effect of students' self-concept of intelligence on the total time spent gazing at peer learners, suggesting that, whereas peer learners' achievement-related behavior (i.e., the hand-raising conditions) did not affect how long students looked at their peers, students who felt more comfortable about their intellectual abilities paid more attention to their virtual classmates. With regard to the IVR configuration—similar to the number of peers looked at and the frequency of gazing at peers (see Tables S1 and S2)—the results revealed that the total time spent gazing at peer learners was statistically significantly higher for participants who were seated in the back of the IVR classroom (d = 1.30) and when the virtual classmates were presented as cartoon-style avatars (d = 0.77). This indicates that with more peer learners in the field of view, participants' visual attention directed toward the peers increased, and participants spent more time processing (or needed more time to process) the social information provided by the peer learners' avatars when the avatars were less realistic.

Table S4 provides detailed statistics and standardized regression coefficients for the effects of the experimental hand-raising conditions on the total time spent gazing at peer learners.

Variables	β	*SE*	*t*	*p*-value	95% CI
(Intercept)	-0.01	0.05	-0.26	0.797	[-0.12, 0.09]
35% vs. 20% hand-raising	-0.02	0.04	-0.40	0.690	[-0.09, 0.06]
65% vs. 20% hand-raising	-0.09	0.08	-1.02	0.308	[-0.25, 0.08]
80% vs. 20% hand-raising	0.05	0.03	1.70	0.089	[-0.01, 0.10]
Back vs. front sitting position	0.48	0.05	10.56	<0.001	[0.39, 0.57]
Realistic vs. cartoon avatars	-0.28	0.03	-10.50	<0.001	[-0.33, -0.23]
Gender (male vs. female)	-0.04	0.05	-0.72	0.469	[-0.14, 0.07]
Math grade	-0.01	0.06	-0.10	0.923	[-0.13, 0.12]
German grade	-0.10	0.08	-1.19	0.234	[-0.07, 0.27]
Prior CT interest	-0.09	0.06	-1.47	0.142	[-0.21, 0.03]
Self-concept of intelligence	0.13	0.03	3.85	<0.001	[0.07, 0.20]
Social orientation	0.08	0.04	1.72	0.086	[-0.01, 0.16]

Table S4. Standardized regression coefficients predicting students' total time spent gazing at peer learners by the experimental hand-raising conditions. *SE* = Standard error; CI = Confidence interval. *p*-values refer to the standardized regression coefficients β. For categorical variables, the second category mentioned serves as the reference category. Grades were on a scale from 1 to 6 with higher numbers indicating lower achievement. Prior CT (Computational Thinking) interest, self-concept of intelligence, and social orientation were assessed on a 4-point rating scale with higher values indicating higher levels of these constructs.

Effects on the mean pupil diameter

Only one of the covariates had an effect on students' mean pupil diameter. The results showed a small negative effect of social orientation, indicating that students with higher levels of social orientation had a lower mean pupil diameter on average during the IVR lesson. A possible explanation for this finding is that students with a high social orientation are used to seeking and processing higher levels of social information and were therefore less affected by the social information provided in the IVR classroom situation.

Table S5 provides detailed statistics and standardized regression coefficients for the effects of the experimental hand-raising conditions on students' mean pupil diameter.

Variables	β	*SE*	*t*	*p*-value	95% CI
(Intercept)	0.00	0.04	-0.09	0.930	[-0.08, 0.07]
35% vs. 20% hand-raising	-0.04	0.05	-0.78	0.433	[-0.14, 0.06]
65% vs. 20% hand-raising	0.05	0.06	0.98	0.328	[-0.06, 0.16]
80% vs. 20% hand-raising	0.20	0.08	2.45	0.014	[0.04, 0.35]
Back vs. front sitting position	-0.04	0.06	-0.69	0.493	[-0.15, 0.07]
Realistic vs. cartoon avatars	0.09	0.06	1.45	0.146	[-0.03, 0.22]
Gender (male vs. female)	0.00	0.04	-0.07	0.946	[-0.07, 0.07]
Math grade	-0.07	0.06	-1.24	0.215	[-0.18, 0.04]
German grade	0.07	0.07	0.89	0.376	[-0.08, 0.21]
Prior CT interest	0.05	0.04	1.35	0.177	[-0.02, 0.12]
Self-concept of intelligence	-0.06	0.06	-0.95	0.344	[-0.18, 0.06]
Social orientation	-0.14	0.07	-1.97	0.048	[-0.29, 0.00]

Table S5. Standardized regression coefficients predicting students' mean pupil diameter by the experimental hand-raising conditions. *SE* = Standard error; CI = Confidence interval. *p*-values refer to the standardized regression coefficients β. For categorical variables, the second category mentioned serves as the reference category. Grades were on a scale from 1 to 6 with higher numbers indicating lower achievement. Prior CT (Computational Thinking) interest, self-concept of intelligence, and social orientation were assessed on a 4-point rating scale with higher values indicating higher levels of these constructs.

Supplementary Appendix 3

Relationships between students' eye movements and their situational self-concept

As described in the Results section, students' self-concept was significantly related to (a) the experimental manipulation of peer learners' hand-raising behavior (particularly comparing the conditions with 20% and 80% hand-raising) and (b) interindividual differences in visual attention to social comparison information (indicated by the number of peers looked at, the frequency of gazing at peers, and the total time spent gazing at peers).

Table S6 below provides detailed statistics and standardized regression coefficients for the regression models predicting students' situational self-concept using the number of peers looked at (Model 1), the frequency of gazing at peers (Model 2), the total time spent gazing at peers (Model 3), and the mean pupil diameter (Model 4). As additional results, Supplementary Appendix 7 provides statistics for the regression models without the full set of covariates.

Variables	*β*	*SE*	*t*	*p*-value	95% CI
Model 1: Number of peers looked at					
(Intercept)	0.00	0.09	-0.02	0.984	[-0.18, 0.18]
Number of students looked at	-0.13	0.05	-2.56	0.010	[-0.23, -0.03]
35% vs. 20% hand-raising	-0.01	0.06	-0.12	0.902	[-0.12, 0.11]
65% vs. 20% hand-raising	-0.07	0.06	-1.11	0.268	[-0.18, 0.05]
80% vs. 20% hand-raising	-0.10	0.04	-2.42	0.015	[-0.19, -0.02]
Back vs. front sitting position	-0.07	0.06	-1.17	0.242	[-0.18, 0.05]
Realistic vs. cartoon avatars	-0.01	0.07	-0.08	0.933	[-0.15, 0.14]
Gender (male vs. female)	0.06	0.10	0.60	0.547	[-0.13, 0.25]
Math grade	-0.12	0.04	-2.70	0.007	[-0.20, -0.03]
German grade	0.20	0.04	5.53	<0.001	[0.13, 0.27]
Prior CT interest	0.04	0.08	0.55	0.580	[-0.11, 0.20]
Self-concept of intelligence	0.13	0.07	1.77	0.077	[-0.01, 0.27]
Social orientation	0.01	0.04	0.33	0.744	[-0.07, 0.10]
Model 2: Frequency of gazing at peers					
(Intercept)	0.00	0.09	0.01	0.992	[-0.18, 0.18]
Frequency of gazing at peers	-0.11	0.05	-2.06	0.040	[-0.21, -0.01]
35% vs. 20% hand-raising	-0.01	0.06	-0.20	0.843	[-0.12, 0.10]
65% vs. 20% hand-raising	-0.07	0.06	-1.13	0.258	[-0.18, 0.05]
80% vs. 20% hand-raising	-0.11	0.05	-2.46	0.014	[-0.20, -0.02]
Back vs. front sitting position	-0.06	0.06	-0.91	0.364	[-0.18, 0.07]
Realistic vs. cartoon avatars	-0.01	0.08	-0.09	0.927	[-0.16, 0.14]
Gender (male vs. female)	0.07	0.10	0.69	0.493	[-0.13, 0.27]
Math grade	-0.11	0.04	-2.68	0.007	[-0.19, -0.03]
German grade	0.19	0.03	5.48	<0.001	[0.12, 0.25]
Prior CT interest	0.05	0.08	0.57	0.571	[-0.11, 0.20]
Self-concept of intelligence	0.13	0.07	1.76	0.078	[-0.01, 0.27]
Social orientation	0.02	0.05	0.39	0.696	[-0.07, 0.11]
Model 3: Total time spent gazing at peers					
(Intercept)	0.00	0.09	-0.01	0.992	[-0.18, 0.18]
Total time spent gazing at peers	-0.10	0.05	-2.27	0.023	[-0.19, -0.01]
35% vs. 20% hand-raising	-0.01	0.06	-0.18	0.860	[-0.12, 0.10]
65% vs. 20% hand-raising	-0.06	0.06	-1.13	0.260	[-0.18, 0.05]
80% vs. 20% hand-raising	-0.11	0.04	-2.50	0.012	[-0.20, -0.02]
Back vs. front sitting position	-0.06	0.06	-0.95	0.341	[-0.17, 0.06]
Realistic vs. cartoon avatars	0.00	0.07	-0.06	0.953	[-0.15, 0.14]
Gender (male vs. female)	0.07	0.10	0.67	0.504	[-0.13, 0.27]
Math grade	-0.11	0.04	-2.68	0.007	[-0.19, -0.03]
German grade	0.19	0.03	5.45	<0.001	[0.12, 0.25]
Prior CT interest	0.05	0.08	0.59	0.556	[-0.11, 0.20]
Self-concept of intelligence	0.13	0.07	1.75	0.080	[-0.02, 0.27]
Social orientation	0.02	0.05	0.34	0.734	[-0.07, 0.11]

(continued)

Variables	*β*	*SE*	*t*	*p*-value	95% CI
Model 4: Mean pupil diameter					
(Intercept)	0.00	0.09	0.00	0.997	[-0.18, 0.18]
Mean pupil diameter	-0.07	0.04	-1.65	0.100	[-0.16, 0.01]
35% vs. 20% hand-raising	-0.01	0.05	-0.22	0.829	[-0.11, 0.09]
65% vs. 20% hand-raising	-0.05	0.06	-0.91	0.361	[-0.16, 0.06]
80% vs. 20% hand-raising	-0.10	0.05	-2.17	0.030	[-0.19, -0.01]
Back vs. front sitting position	-0.11	0.06	-1.72	0.086	[-0.23, 0.02]
Realistic vs. cartoon avatars	-0.03	0.07	0.44	0.661	[-0.11, 0.17]
Gender (male vs. female)	0.07	0.10	0.70	0.484	[-0.13, 0.27]
Math grade	-0.12	0.04	-2.64	0.008	[-0.20, -0.03]
German grade	0.18	0.04	5.31	<0.001	[0.12, 0.25]
Prior CT interest	0.06	0.08	0.75	0.451	[-0.09, 0.21]
Self-concept of intelligence	0.11	0.07	1.52	0.128	[-0.03, 0.26]
Social orientation	0.00	0.05	-0.01	0.995	[-0.10, 0.09]

Table S6. Standardized regression coefficients predicting students' situational self-concept from their eye movements. *SE* = Standard error; CI = Confidence interval. *p*-values refer to the standardized regression coefficients *β*. For categorical variables, the second category mentioned serves as the reference category. Grades were on a scale from 1 to 6 with higher numbers indicating lower achievement. Prior CT (Computational Thinking) interest, self-concept of intelligence, and social orientation were assessed on a 4-point rating scale with higher values indicating higher levels of these constructs.

Beyond these findings, the results showed that students' grades in mathematics and German were statistically significant predictors of their situational self-concept in all three regression models using the different eye movements as predictors. More specifically, the results revealed that better grades in mathematics led to a higher situational self-concept regarding the IVR lesson on computational thinking, whereas better grades in German were associated with lower situational self-concept. These results indicate that, whereas students' active social comparisons in the IVR situation—as indicated by the time they spent looking at their peer learners and their mean pupil diameter—had an effect on how they evaluated themselves, students seemed to also base their self-evaluations on their achievement in other (dis)similar subjects.

Supplementary Appendix 4

Details on the ray-casting technique used to identify objects of gaze in the IVR

In an IVR set-up as in the present study, the eye-tracking device is not stationary as it is in traditional eye-tracking experiments. Rather, it is part of the HMD, which can be moved 360 degrees in the virtual space. To identify the object of gaze, we implemented an algorithm to apply ray-casting[1,2], a technique typically used to calculate gaze points from eye-tracking devices[3]. The idea of gaze ray-casting is to forward a person's 3D gaze vector into the virtual environment and to identify which virtual object the gaze hits[4]. Ray casting was performed for every measured time point during the virtual experiment (i.e., on average, every 24 milliseconds, depending on hardware and software performance). Using predefined functions from the Unreal Engine Blueprint (SetActorLocation, SetActorRotation and GetRotationXVector)[5], participants' gaze direction in the environment was calculated as a vector combination of their head position, head direction, and gaze offset starting from the person's position in the VR environment. From this position, their head direction was calculated as a vector that was orthogonal to the screen surface of the HMD pointing from their head into the environment. Because this orthogonal vector reflects the head but not gaze direction, we needed to rotate this vector to reflect participants' actual gaze direction. Participants' relative gaze direction, measured with the integrated eye tracker, was then used to rotate the head direction such that it reflected the absolute gaze direction of the person pointing into the virtual environment. To calculate this rotation, we used pitch (i.e., the angle at which one is looking up or down) and yaw (i.e., the rotation of one's head left or right from a vertical axis) as two markers describing the orientation in the 3D space. We then calculated the angles between the combined, normalized gaze vector and the x coordinate of the local space—both given in local coordinates because the integrated eye tracker has no information about the position or orientation of the HMD in the virtual space. Based on the general calculation of an angle (in degrees) of two vectors (v_1, v_2) as

$$\alpha = \arccos\left(\frac{v_1 \cdot v_2}{|v_1| \cdot |v_2|}\right) \cdot \frac{180}{\pi}.$$

We calculated the yaw rotation as the angle between the x vector $x = (1\ 0\ 0)^T$ and the flat gaze vector $g_{flat} = (g_1\ g_2\ 0)^T$. We calculated the pitch rotation as the angle between the gaze vector $g = (g_1\ g_2\ g_3)^T$ and g_{flat}. The global gaze vector we used was calculated as a vector that was the sum of the head position and the lengthened head orientation vector pointing into the environment after it was rotated by the calculated gaze angles pitch and yaw.

To perform the ray-casting, we additionally needed to extract information about the position and shape of each virtual object to ultimately calculate when it would be hit. We therefore added colliders to the objects in the IVR (i.e., an invisible mesh grid that approximates the shape of an object and describes its surface). The colliders could be used to detect gaze hit. To obtain the object of gaze across the full experimental session, we applied ray-casting frame by frame for the entire IVR lesson for each participant. We counted a gaze hit on an object if

$$\{v_{location} + (v_{direction} \cdot k) | k \in \mathbb{R}\} \cap s_{object} \neq \emptyset$$

where $v_{location} \in \mathbb{R}^3$ describes the coordinates of a person's eye location, $v_{direction} \in \mathbb{R}^3$ describes the normalised combined gaze direction (i.e., the equidistant line between the gaze direction of the left and right eyes), and s_{object} reflects the set of coordinates describing the surface of the object. We used the LineTraceByChannel function from the Unreal Engine Blueprint[5], which outputted the name of the object hit by the ray-cast gaze vector for each frame. We used these values to calculate the object of interest information (i.e., frequency of gazing at virtual classmates, total time spent gazing at virtual classmates). Notably, in our case, only gaze and head information were collected during the experiment, and we applied the ray-casting algorithm afterward using a C++ script to map the collected eye-tracking data onto the Unreal Engine while rerunning the entire IVR lesson for each participant according to the tracked time stamps.

References

1. Pietroszek, K. Raycasting in virtual reality in *Encyclopedia of Computer Graphics and Games* (ed. Lee, N.) 1–3 (Springer, 2018).
2. Roth, S. D. Ray casting for modeling solids. *Comput. Graph. Image Process.* **18**, 109–144 (1982).
3. Tobii Tech. What is eye tracking? https://tech.tobii.com/technology/what-is-eye-tracking/ (2020).
4. Soret, R., Charras, P., Khazar, I., Hurter, C. & Peysakhovich, V. Eye-tracking and virtual reality in 360-degrees: Exploring two ways to assess attentional orienting in rear space. *Symp. Eye Track. Res. Appl.* 1–7 (2020) doi:10.1145/3379157.3391418.
5. Epic Games. Blueprints visual scripting. https://docs.unrealengine.com/en-US/Engine/Blueprints/index.html (2020).

Supplementary Appendix 5

Correlation matrix for the eye-movement features

Table S7 provides detailed statistics and standardized regression coefficients for the effects of the experimental hand-raising conditions on students' mean pupil diameter.

Variable	1	2	3	4	5	6	7	8	9	10
1. Number of peers looked at	—									
2. Frequency of gazing at peers	0.70***<br>[0.63, 0.76]	—								
3. Total time spent gazing at peers	0.67<br>[0.60, 0.73]	0.99***<br>[0.98, 0.99]	—							
4. Mean pupil diameter	-0.01<br>[-0.13, 0.11]	-0.04<br>[-0.16, 0.08]	-0.06<br>[-0.18, 0.06]	—						
5. Gender	-0.06<br>[-0.18, 0.06]	0.00<br>[-0.12, 0.12]	-0.01<br>[-0.13, 0.11]	0.08<br>[-0.04, 0.20]	—					
6. Math grade	-0.01<br>[-0.13, 0.11]	-0.03<br>[-0.15, 0.09]	-0.04<br>[-0.16, 0.08]	-0.03<br>[-0.15, 0.09]	-0.02<br>[-0.12, 0.09]	—				
7. German grade	0.03<br>[-0.09, 0.15]	0.00<br>[-0.12, 0.12]	-0.01<br>[-0.13, 0.11]	-0.01<br>[-0.13, 0.11]	0.23***<br>[0.12, 0.33]	0.55<br>[0.47, 0.62]	—			
8. Prior CT interest	-0.10<br>[-0.22, 0.02]	-0.11<br>[-0.23, 0.01]	-0.10<br>[-0.22, 0.02]	0.05<br>[-0.07, 0.17]	0.38***<br>[0.29, 0.47]	0.03<br>[-0.08, 0.14]	0.14**<br>[0.03, 0.24]	—		
9. Self-concept of intelligence	0.09<br>[-0.03, 0.21]	0.11<br>[-0.01, 0.26]	0.11<br>[-0.01, 0.23]	-0.07<br>[-0.19, 0.05]	0.07<br>[-0.03, 0.17]	-0.32***<br>[-0.41, -0.22]	-0.31***<br>[-0.40, -0.21]	0.00<br>[-0.11, 0.11]	—	
10. Social orientation	-0.02<br>[-0.13, 0.10]	0.00<br>[-0.12, 0.12]	-0.01<br>[-0.13, 0.11]	-0.11<br>[-0.23, 0.01]	-0.09<br>[-0.19, 0.01]	0.02<br>[-0.09, 0.13]	0.00<br>[-0.11, 0.11]	0.06<br>[-0.05, 0.16]	-0.15**<br>[-0.25, -0.05]	—

Table S7. Variables 1-9 are non-normally distributed; thus, Spearman's rho is reported. 95% confidence intervals are given in brackets. Gender 1 = female, 2 = male. Grades were on a scale from 1 to 6 with higher numbers indicating lower achievement. Prior CT (Computational Thinking) interest, self-concept of intelligence, and social orientation were assessed on a 4-point rating scale with higher values indicating higher levels of these constructs. ** *p*-values < 0.01; *** *p*-values < 0.001.

Supplementary Appendix 6

Additional analyses: Effects of virtual peers' hand raising on students' eye movements (without covariates)

In the following, detailed statistics and standardized regression coefficients for the effects of the experimental hand-raising conditions on the different eye-movement features (i.e., the number of peer learners looked at, the frequency of gazing at peer learners, the total time spent gazing at peer learners, and the mean pupil diameter) are provided. Supplementary Appendix 2 provides detailed statistics for the respective regression models including the full set of covariates as reported in the paper.

Effects on the number of peer learners looked at

Table S8 below provides detailed statistics and standardized regression coefficients for the effects of the experimental hand-raising conditions on the number of peer learners looked at.

	Model 1: Without covariates					Model 2: Controlling only for IVR configurations				
Variables	*β*	*SE*	*t*	*p*-value	95% CI	*β*	*SE*	*t*	*p*-value	95% CI
(Intercept)	-0.01	0.11	-0.01	0.925	[-0.22, 0.20]	-0.01	0.08	-0.11	0.915	[-0.16, 0.14]
35% vs. 20% hand-raising	-0.01	0.08	-0.14	0.890	[-0.17, 0.15]	0.01	0.07	0.11	0.911	[-0.12, 0.14]
65% vs. 20% hand-raising	-0.08	0.08	-0.96	0.338	[-0.24, 0.08]	-0.05	0.08	-0.60	0.546	[-0.20, 0.10]
80% vs. 20% hand-raising	0.09	0.05	1.76	0.048	[0.00, 0.18]	0.08	0.04	1.76	0.048	[0.00, 0.16]
Back vs. front sitting position						0.30	0.06	5.43	<0.001	[0.19, 0.41]
Realistic vs. cartoon avatars						-0.22	0.06	-3.67	<0.001	[-0.34, -0.10]

Table S8. Standardized regression coefficients predicting the number of peer learners looked at by the experimental hand-raising conditions. *SE* = Standard error; CI = Confidence interval. *p*-values refer to the standardized regression coefficients *β*. For categorical variables, the second category mentioned serves as the reference category.

Effects on the frequency of gazing at peer learners

Table S9 below provides detailed statistics and standardized regression coefficients for the effects of the experimental hand-raising conditions on the frequency of gazing at peer learners.

	Model 1: Without covariates					Model 2: Controlling only for IVR configurations				
Variables	*β*	*SE*	*t*	*p*-value	95% CI	*β*	*SE*	*t*	*p*-value	95% CI
(Intercept)	-0.01	0.10	-0.11	0.913	[-0.21, 0.18]	-0.01	0.07	-0.08	0.935	[-0.13, 0.12]
35% vs. 20% hand-raising	-0.06	0.07	-0.78	0.438	[-0.20, 0.09]	-0.03	0.04	-0.78	0.437	[-0.10, 0.05]
65% vs. 20% hand-raising	-0.11	0.09	-1.18	0.238	[-0.29, 0.07]	-0.06	0.08	-0.80	0.426	[-0.21, 0.09]
80% vs. 20% hand-raising	0.06	0.04	1.53	0.125	[-0.02, 0.13]	0.04	0.03	1.26	0.209	[0.00, 0.10]
Back vs. front sitting position						0.47	0.05	9.45	<0.001	[0.37, 0.56]
Realistic vs. cartoon avatars						-0.29	0.03	-9.12	<0.001	[-0.36, -0.23]

Table S9. Standardized regression coefficients predicting students' frequency of gazing at peer learners by the experimental hand-raising conditions. *SE* = Standard error; CI = Confidence interval. *p*-values refer to the standardized regression coefficients *β*. For categorical variables, the second category mentioned serves as the reference category.

Effects on the total time spent gazing at peer learners

Table S10 provides detailed statistics and standardized regression coefficients for the effects of the experimental hand-raising conditions on the total time spent gazing at peer learners.

	Model 1: Without covariates					Model 2: Controlling only for IVR configurations				
Variables	*β*	*SE*	*t*	*p*-value	95% CI	*β*	*SE*	*t*	*p*-value	95% CI
(Intercept)	-0.01	0.10	-0.11	0.914	[-0.20, 0.18]	0.0	0.06	-0.06	0.954	[-0.12, 0.11]
35% vs. 20% hand-raising	-0.05	0.08	-0.59	0.553	[-0.20, 0.11]	-0.02	0.04	-0.46	0.646	[-0.09, 0.06]
65% vs. 20% hand-raising	-0.11	0.10	-1.15	0.251	[-0.30, 0.08]	-0.06	0.08	-0.76	0.445	[-0.22, 0.10]
80% vs. 20% hand-raising	0.06	0.05	1.23	0.220	[-0.03, 0.14]	0.04	0.04	0.98	0.328	[-0.04, 0.10]
Back vs. front sitting position						0.49	0.05	9.69	<0.001	[0.39, 0.59]
Realistic vs. cartoon avatars						-0.28	0.03	-8.37	<0.001	[-0.35, -0.22]

Table S10. Standardized regression coefficients predicting students' total time spent gazing at peer learners by the experimental hand-raising conditions. *SE* = Standard error; CI = Confidence interval. *p*-values refer to the standardized regression coefficients *β*. For categorical variables, the second category mentioned serves as the reference category.

Effects on the mean pupil diameter

Table S11 provides detailed statistics and standardized regression coefficients for the effects of the experimental hand-raising conditions on students' mean pupil diameter.

	Model 1: Without covariates					Model 2: Controlling only for IVR configurations				
Variables	*β*	*SE*	*t*	*p*-value	95% CI	*β*	*SE*	*t*	*p*-value	95% CI
(Intercept)	-0.01	0.04	-0.31	0.756	[-0.10, 0.07]	-0.01	0.04	-0.25	0.804	[-0.09, 0.07]
35% vs. 20% hand-raising	-0.04	0.06	-0.66	0.513	[-0.15, 0.07]	-0.04	0.06	-0.71	0.475	[-0.15, 0.07]
65% vs. 20% hand-raising	0.04	0.05	0.88	0.378	[-0.05, 0.13]	0.03	0.05	0.70	0.487	[-0.06, 0.12]
80% vs. 20% hand-raising	0.21	0.08	2.60	0.009	[0.05, 0.36]	0.21	0.08	2.60	0.009	[0.05, 0.36]
Back vs. front sitting position						-0.03	0.05	-0.66	0.507	[-0.13, 0.06]
Realistic vs. cartoon avatars						0.10	0.06	1.56	0.122	[-0.03, 0.22]

Table S11. Standardized regression coefficients predicting students' mean pupil diameter by the experimental hand-raising conditions. *SE* = Standard error; CI = Confidence interval. *p*-values refer to the standardized regression coefficients *β*. For categorical variables, the second category mentioned serves as the reference category.

Supplementary Appendix 7

Additional analyses: Relationships between students' eye movements and their situational self-concept (without covariates)

Table S12 below provides detailed statistics and standardized regression coefficients for the regression models predicting students' situational self-concept using the number of peers looked at (Model 1), the frequency of gazing at peers (Model 2), the total time spent gazing at peers (Model 3), and the mean pupil diameter (Model 4). Supplementary Appendix 3 provides detailed statistics for the respective regression models including the full set of covariates as reported in the paper.

	Models without covariates					Models controlling only for IVR configurations				
Variables	β	*SE*	*t*	*p*-value	95% CI	β	*SE*	*t*	*p*-value	95% CI
Model 1: Number of peers looked at										
(Intercept)	0.00	0.10	-0.01	0.990	[-0.20, 0.19]	0.00	0.10	-0.01	0.992	[-0.19, 0.19]
Number of students looked at	-0.17	0.07	-2.48	0.013	[-0.30, -0.04]	-0.14	0.07	-2.07	0.038	[-0.27, -0.01]
35% vs. 20% hand-raising	-0.01	0.06	-0.24	0.811	[-0.13, 0.10]	-0.02	0.06	-0.28	0.782	[-0.13, 0.10]
65% vs. 20% hand-raising	-0.05	0.06	-0.86	0.390	[-0.17, 0.07]	-0.05	0.06	-0.86	0.393	[-0.17, 0.07]
80% vs. 20% hand-raising	-0.10	0.05	-2.02	0.043	[-0.20, 0.00]	-0.10	0.05	-2.11	0.035	[-0.20 -0.01]
Back vs. front sitting position						-0.08	0.06	-1.37	0.170	[-0.18, 0.03]
Realistic vs. cartoon avatars						-0.03	0.06	-0.41	0.685	[-0.15, 0.10]
Model 2: Frequency of gazing at peers										
(Intercept)	0.00	0.10	0.01	0.990	[-0.19, 0.19]	0.00	0.10	-0.01	0.996	[-0.19, 0.19]
Frequency of gazing at peers	-0.15	0.06	-2.34	0.019	[-0.27, -0.02]	-0.10	0.06	-1.82	0.049	[-0.22, 0.00]
35% vs. 20% hand-raising	-0.02	0.06	-0.38	0.705	[-0.13, 0.09]	-0.02	0.05	-0.39	0.700	[-0.13, 0.08]
65% vs. 20% hand-raising	-0.06	0.06	-0.99	0.322	[-0.17, 0.06]	-0.05	0.06	-0.92	0.358	[-0.16, 0.06]
80% vs. 20% hand-raising	-0.11	0.05	-2.10	0.036	[-0.21, -0.01]	-0.11	0.05	-2.20	0.028	[-0.21, -0.01]
Back vs. front sitting position						-0.07	0.06	-1.17	0.242	[-0.18, 0.05]
Realistic vs. cartoon avatars						-0.03	0.07	-0.38	0.701	[-0.16, 0.11]
Model 3: Total time spent gazing at peers										
(Intercept)	0.00	0.10	-0.01	0.991	[-0.19, 0.19]	0.00	0.10	0.00	0.998	[-0.19, 0.19]
Total time spent gazing at peers	-0.15	0.06	-2.30	0.022	[-0.27, -0.02]	-0.10	0.05	-1.87	0.031	[-0.21, 0.00]
35% vs. 20% hand-raising	-0.02	0.06	-0.35	0.726	[-0.13, 0.09]	-0.02	0.05	-0.37	0.715	[-0.12, 0.08]
65% vs. 20% hand-raising	-0.06	0.06	-1.00	0.320	[-0.17, 0.06]	-0.05	0.06	-0.92	0.357	[-0.16, 0.06]
80% vs. 20% hand-raising	-0.11	0.05	-2.13	0.033	[-0.21, -0.01]	-0.11	0.05	-2.23	0.026	[-0.21, -0.01]
Back vs. front sitting position						-0.07	0.06	-1.23	0.221	[-0.17, 0.04]
Realistic vs. cartoon avatars						-0.02	0.07	-0.36	0.719	[-0.15, 0.11]

(continued)

	Models without covariates					Models controlling only for IVR configurations				
Model 4: Mean pupil diameter										
(Intercept)	0.00	0.10	-0.01	0.995	[-0.20, 0.20]	0.00	0.10	-0.01	0.996	[-0.19, 0.19]
Mean pupil diameter	-0.05	0.04	-1.12	0.261	[-0.14, 0.04]	-0.06	0.05	-1.19	0.233	[-0.16, 0.04]
35% vs. 20% hand-raising	-0.02	0.05	-0.31	0.756	[-0.13, 0.09]	-0.02	0.05	-0.41	0.682	[-0.12, 0.08]
65% vs. 20% hand-raising	-0.04	0.06	-0.63	0.529	[-0.16, 0.08]	-0.04	0.06	-0.79	0.431	[-0.16, 0.07]
80% vs. 20% hand-raising	-0.11	0.06	-1.90	0.047	[-0.22, 0.00]	-0.10	0.05	-2.01	0.044	[-0.20, 0.00]
Back vs. front sitting position						-0.12	0.06	-1.97	0.058	[-0.24, 0.00]
Realistic vs. cartoon avatars						0.01	0.07	0.15	0.882	[-0.12, 0.14]

Table S12. Standardized regression coefficients predicting students' situational self-concept from their eye movements. *SE* = Standard error; CI = Confidence interval. *p*-values refer to the standardized regression coefficients *β*. For categorical variables, the second category mentioned serves as the reference category.
